# Mapping three-dimensional intratumor proteomic heterogeneity in uterine serous carcinoma by multiregion microsampling

**DOI:** 10.1186/s12014-024-09451-2

**Published:** 2024-01-22

**Authors:** Allison L. Hunt, Nicholas W. Bateman, Waleed Barakat, Sasha C. Makohon-Moore, Tamara Abulez, Jordan A. Driscoll, Joshua P. Schaaf, Brian L. Hood, Kelly A. Conrads, Ming Zhou, Valerie Calvert, Mariaelena Pierobon, Jeremy Loffredo, Katlin N. Wilson, Tracy J. Litzi, Pang-Ning Teng, Julie Oliver, Dave Mitchell, Glenn Gist, Christine Rojas, Brian Blanton, Kathleen M. Darcy, Uma N. M. Rao, Emanuel F. Petricoin, Neil T. Phippen, G. Larry Maxwell, Thomas P. Conrads

**Affiliations:** 1 Women’s Health Integrated Research Center, Inova Women’s Service Line, Inova Health System, 3289 Woodburn Rd, Suite 375, Annandale, VA 22042 USA; 2 Gynecologic Cancer Center of Excellence and the Women’s Health Integrated Research Center, Department of Gynecologic Surgery and Obstetrics, Uniformed Services University of the Health Sciences, Walter Reed National Military Medical Center, 8901 Wisconsin Avenue, Bethesda, MD 20889 USA; 3 The Henry M. Jackson Foundation for the Advancement of Military Medicine, Inc, 6720A Rockledge Drive, Suite 100, Bethesda, MD 20817 USA; 4 Department of Surgery, The John P. Murtha Cancer Center Research Program, Uniformed Services University of the Health Sciences, Walter Reed National Military Medical Center, 8901 Wisconsin Avenue, Bethesda, MD 20889 USA; 5 Center for Applied Proteomics and Molecular Medicine, George Mason University, Manassas, VA USA

**Keywords:** Uterine serous carcinoma, Proteomics, Proteogenomics, Laser microdissection, Tumor microenvironment, Intratumor heterogeneity, Spatial proteomics

## Abstract

**Background:**

Although uterine serous carcinoma (USC) represents a small proportion of all uterine cancer cases, patients with this aggressive subtype typically have high rates of chemotherapy resistance and disease recurrence that collectively result in a disproportionately high death rate. The goal of this study was to provide a deeper view of the tumor microenvironment of this poorly characterized uterine cancer variant through multi-region microsampling and quantitative proteomics.

**Methods:**

Tumor epithelium, tumor-involved stroma, and whole “bulk” tissue were harvested by laser microdissection (LMD) from spatially resolved levels from nine USC patient tumor specimens and underwent proteomic analysis by mass spectrometry and reverse phase protein arrays, as well as transcriptomic analysis by RNA-sequencing for one patient’s tumor.

**Results:**

LMD enriched cell subpopulations demonstrated varying degrees of relatedness, indicating substantial intratumor heterogeneity emphasizing the necessity for enrichment of cellular subpopulations prior to molecular analysis. Known prognostic biomarkers were quantified with stable levels in both LMD enriched tumor and stroma, which were shown to be highly variable in bulk tissue. These USC data were further used in a comparative analysis with a data generated from another serous gynecologic malignancy, high grade serous ovarian carcinoma, and have been added to our publicly available data analysis tool, the Heterogeneity Analysis Portal (https://lmdomics.org/).

**Conclusions:**

Here we identified extensive three-dimensional heterogeneity within the USC tumor microenvironment, with disease-relevant biomarkers present in both the tumor and the stroma. These data underscore the critical need for upfront enrichment of cellular subpopulations from tissue specimens for spatial proteogenomic analysis.

**Supplementary Information:**

The online version contains supplementary material available at 10.1186/s12014-024-09451-2.

## Introduction

Cancer-related deaths have declined in the United States since 1990, though the incidence and mortality of specific malignancies such as uterine cancer have increased, with projections estimating 66,200 new uterine carcinoma cases and 13,030 related deaths in 2023 [1]. Uterine serous carcinoma (USC) represents only a small proportion of all uterine cancer cases, but patients with this aggressive subtype typically have high rates of chemotherapy resistance, disease recurrence, and constitute a disproportionately high percentage of the deaths, owing in part to significant heterogeneity present in the tumor microenvironment (TME) [2–4].

The guidelines for staging USC have recently incorporated results from molecular analyses, including evaluation of microsatellite satellite instability (MSI), mismatch repair (MMR), *HER2* overexpression and/or gene amplification, loss of *PTEN*, copy number variations (CNV), and the detection of mutations in *TP53*, *PIK3CA*, *AKT*, *MAPK*, and *POLE* [5–8]. Standard first-line clinical management of USC relies on surgical staging with consideration of maximal tumor debulking for gross disease, followed by administration of systemic adjuvant platinum and taxane-based therapies with/without external beam radiation therapy (EBRT) and/or vaginal cuff brachytherapy (VBT) [8]. Trastuzumab is additionally recommended for patients with stage III/IV HER2-positive disease [9]. Recent clinical trials have additionally demonstrated significantly improved outcomes for endometrial cancer patients when chemotherapy is given in combination with pembrolizumab [10], pembrolizumab plus lenvatinib [11], or dostarlimab [12], with further differences observed in the NRG-GY018 (pembrolizumab plus chemotherapy [10]) and RUBY (dostarlimab plus chemotherapy [12]) trials depending on MMR proficiency.

To improve our understanding of intratumor heterogeneity (ITH) within the USC TME, we investigated proteome and transcriptome alterations in spatially resolved laser microdissection (LMD) enriched cellular subpopulations from nine USC patient tumor tissue specimens. LMD enriched samples were analyzed by liquid chromatography-tandem mass spectrometry (LC–MS/MS) and reverse phase protein microarray (RPPA), and one patient’s tumor was further analyzed by targeted RNA-sequencing (RNA-seq). Here we identified extensive intratumor heterogeneity in primary tumors from USC patients, emphasizing the value of enrichment of cellular subpopulations prior to molecular analysis for a more selective molecular view of the TME. We additionally highlight several similarities and differences between the proteomic profiles of LMD enriched cellular subpopulations from USC patients with a similar TME-resolved sample set from high grade serous ovarian carcinoma (HGSOC) patient tumor specimens [13].

## Methods

### Tissue specimens

Surgically resected fresh-frozen tissue specimens embedded in optimal cutting temperature (O.C.T.) compound were obtained from nine patients with stage II (n = 1; patient 343WD) or III (n = 8) USC (Additional file 8: Table S1). All tissues used in this study were obtained from the primary site of disease, with the exception of the specimen from patient 343WE, from whom a metastatic lesion from the ovary was obtained. Eight of the nine patients were chemotherapy-naïve at the time of surgical resection and specimen acquisition; patient 343WF received neoadjuvant chemotherapy (NACT). All study protocols were approved for use under a Western IRB-approved protocol “An Integrated Molecular Analysis of Endometrial and Ovarian Cancer to Identify and Validate Clinically Informative Biomarkers” deemed exempt under US Federal regulation 45 CFR 46.102(f). All experimental protocols involving human data in this study were in accordance with the Declaration of Helsinki and informed consent was obtained from all patients.

The specimen blocks were sectioned by cryotome into 110–220 consecutive 10 µm thin tissue Sects. (1.1–2.2 mm total depth), depending on tissue availability to avoid complete exhaustion of the specimen block. Tissue sections were placed on polyethylene naphthalate (PEN) membrane slides (Leica Microsystems). Representative sections after every 10 PEN membrane slide Sects. (100 µm) were mounted on charged glass slides and stained with hematoxylin and eosin (H&E).

### Laser microdissection

The slides from each specimen block were separated into five equally sized and spatially distinct regions (levels) assigned by depth within the block, H&E stained, and laser microdissected (LMD7, Leica Microsystems) for analysis via liquid chromatography-tandem mass spectrometry (LC–MS/MS), reverse phase protein microarray (RPPA), and RNA sequencing (RNA-seq; patient 343VY only), as previously described [13]. Briefly, LMD was used to isolate distinct cellular subpopulations of enriched tumor epithelium (ET) or tumor-involved stroma (ES) from slide sections throughout the 5 separate spatially distinct levels of the specimen block. For each of the 5 levels, adjacent interlaced slides were used for whole (“bulk”) tissue (BT) harvests for the collection of all available tissue per section, with the exclusion of necrosis, blood, and adipose tissue. Slides designated for MS proteomics (n = 9 patients), RPPA (n = 9 patients), and transcriptomics (n = 1 patient) within each level were interlaced as much as possible. Within each level per specimen block, LMD was used to enrich cross-sectional areas of 40 mm^2^ and 15 mm^2^ (ET, ES, and BT) for analysis via LC–MS/MS and RPPA, respectively. For case 343VY, cross-sectional areas of 25 mm^2^ of ET, ES, and BT were harvested for RNA-seq. Representative images before and after LMD were captured using the Aperio AT2 slide scanner (Leica Microsystems).

### Peptide preparation and TMT liquid chromatography-tandem mass spectrometry

LMD tissue underwent pressure-assisted, trypsin-digestion, and 5 µg of tryptic peptides per sample were labeled with isobaric Tandem Mass Tags (TMTpro 16plex, ThermoFisher Scientific, Inc.) as previously described [13]. Samples were organized in patient-specific TMT multiplexes and fractionated by basic reversed-phase liquid chromatography (bRPLC). A patient-specific reference pooled sample was generated from all ET, ES, and BT samples from the patient for a given multiplex. Additional patient-specific BT pool samples were generated to fill TMT channels that would have otherwise been filled by samples with < 5 µg of peptide digest and were incorporated into each multiplex as needed. Samples were pooled to generate 24 concatenated fractions, each of which were analyzed by LC–MS/MS using a nanoflow LC system (EASY-nLC 1200, ThermoFisher Scientific, Inc.) coupled online with a Q Exactive HF-X MS (ThermoFisher Scientific, Inc.), as previously described [13]. Briefly, each sample was loaded onto a nanoflow HPLC system outfitted with a reversed-phase trap column (Acclaim PepMap100 C18, 2 cm, nanoViper; ThermoFisher Scientific, Inc.) and a heated (50 ℃) reversed-phase analytical column (Acclaim PepMap RSLC C18, 2 µm, 100 Å, 75 μm × 500 mm, nanoViper; ThermoFisher Scientific, Inc.). Peptides were eluted by developing a linear gradient of 2% mobile phase B (95% acetonitrile with 0.1% formic acid) to 32% mobile phase B within 120 min at a constant flow rate of 250 nL/min. MS1 and MS2 spectra were collected in profile mode, S-lens RF level was set to 40 and voltage was set at 2 kV. MS1 parameters: resolution, 60,000 at *m/z* 200; mass range, *m/z* 400 – 1,600; AGC, 3e6; maximum IT, 45 ms. MS2 parameters: loop count, 12; resolution, 45,000 at *m/z* 200; AGC, 1e5; maximum IT, 95 ms; isolation window, *m/*z 1.0; isolation offset, *m/*z 0.2; fixed first mass, *m/*z 100; charge state, 2–4; intensity threshold, 2e5; nce, 30; dynamic exclusion, 20 s. High resolution (R = 60,000 at *m/z* 200) broadband (*m/z* 400–1600) mass spectra (MS) were acquired from which the top 12 most intense molecular ions in each MS scan were selected for high-energy collisional dissociation (HCD, normalized collision energy of 34) acquisition in the orbitrap at high resolution (R = 45,000 at *m/z* 200). Peptide identification, normalization, and protein-level quantitation using patient-specific imputations was performed as previously described [14]. Briefly, global protein-level abundances were generated from peptide spectral matches (PSM) identified by searching.raw files with a publicly-available, non-redundant human proteome database (http://www.uniprot.org/, SwissProt, Homo sapiens, downloaded 12–01-2017) using Mascot (Matrix Science, v2.6.0), Proteome Discoverer (v2.2.0.388, Thermo FisherScientific, Inc., Waltham, MA, USA), and in-house tools using identical parameters. The.raw data files corresponding to each LC–MS/MS injection per TMTpro16 multiplex were searched using the following parameters: precursor mass tolerance of 10 ppm, fragment ion tolerance of 0.05 Da, a maximum of two tryptic miscleavages, static modification for TMT reporter ion tags (304.2071 Da) on N-termini and lysyl residues, and dynamic modifications for oxidation (15.9949 Da) on methionine residues. The resulting peptide spectral matches (PSMs) were filtered using a false-discovery rate (FDR) < 1.0% (q-value < 0.01), as determined by the Percolator [15] module of Proteome Discoverer. Quan correction was applied to all reagent ion abundances using TMTpro16 reagent lot UL297970. PSMs lacking a TMT reporter ion signal in TMT channel m/z 126 (TMT-126, the patient-specific pooled reference sample combined from all sample digests for a given patient), PSMs lacking TMT reporter ion intensity in all TMT channels, or PSMs exhibiting an isolation interference of ≥ 50% were excluded from downstream analyses. Protein-level abundances were calculated from normalized, median log_2_-transformed TMT reporter ion ratio abundances from a minimum of two PSMs corresponding to a single protein accession. Normalized log_2_-transformed protein-level abundances for each TMTpro16 multiplex were merged and protein-level abundance for proteins not quantified in all patient samples, but in at least ≥ 50%, were imputed using a k-nearest neighbor (k-NN) neighbor strategy using the impute R-package [16]. QA/QC standards were analyzed before and after every sample multiplex to confirm low technical variability (5.4% RSD in PSM counts).

### RNA sequencing

Samples for RNA-seq from patient 343VY were prepared as previously described [13]. Briefly, cells were harvested by LMD into Buffer RLT with β-mercaptoethanol and RNA isolated using the RNeasy Micro Kit (Qiagen) per the manufacturer’s instructions. RNA concentrations were determined by fluorescence (Qubit HS and BR kits, ThermoFisher Scientific, Inc.). RNA integrity numbers (RIN) were determined using the RNA 6000 Pico Kit 2100 Bioanalyzer (Agilent Technologies, Inc.); RIN values were > 6 for all levels and collection types. RNA samples were reverse transcribed to generate barcoded cDNA libraries that were sequenced on the Ion Torrent S5 XL (ThermoFisher Scientific, Inc.). Barcoded cDNA libraries contained 6 LMD RNA samples, a Universal Human Reference RNA (UHR) standard (Stratagene), and a no-template control (NTC) water blank. Successful sequencing runs achieved an average of 18 M reads/sample (with one exception) and 167-205X AQ20 mean coverage depth.

### Reverse phase protein microarray

ET, ES, and BT lysates in the RPPA extraction/lysis buffer were boiled and used for microarray printing onto nitrocellulose-coated glass slides (Grace Bio-Labs) in technical triplicates using a 2470 Aushon Arrayer (Aushon BioSystems, Inc.), as previously described [17, 18]. Briefly, selected arrays were stained with Sypro Ruby Protein Blot Stain (Invitrogen) to assess the amount of protein in each sample for normalization purposes. Prior to antibody staining, the arrays were first treated with Reblot Antibody Stripping solution (Millipore), washed with PBS, and incubated with I-block (Applied Biosystems). To reduce unspecific binding between endogenous proteins and the detection system, arrays were then probed with 3% hydrogen peroxide, an avidin/biotin blocking system (Dako Cytomation), and an additional serum free protein block (Dako Cytomation) using an automated system (Dako Cytomation). Arrays were probed with 281 antibodies targeting native and/or post-translationally modified proteins with known relevance to gynecologic cancers, co-arrayed in technical triplicates using the same antibodies as previously published in Hunt et al. Additional file 8: Table S18 [13]. The selected antibodies were validated previously by Western blot to confirm sensitivity and specificity, specifically to confirm the presence of a single band of correct molecular weight in the positive control sample and the absence of a band in the negative control sample. Antibodies targeting phosphorylated epitopes were further validated by ligand induction, and a subset of antibodies was additionally validated against peptide competition. Each array was printed with a series of positive and negative control cell lysates derived from living cells under cell culture conditions wherein the cells were exposed to a known ligand (e.g. EGF) or chemical agent (e.g. pervanadate) that stimulates the activation (phosphorylation) of a known protein, or the lysate was derived from cell lines with known and verified expression of the specific protein being measured (e.g. HER2) [17, 18]. Signal amplification was achieved using a tyramide-based avidin/biotin amplification system (Dako Cytomation) coupled with the fluorescent IRDye680 dye (LI-COR Biosciences), per manufacturer’s instructions. Arrays were imaged using a laser scanner (TECAN) and analyzed using the MicroVigene software (Vigenetech). Each sample was normalized to the corresponding amount of protein derived from Sypro Ruby stained slides and the triplicates were averaged.

### Quantification and statistical analyses

Bioinformatic and statistical analyses were performed as previously described [13]. Briefly, unsupervised hierarchical clustering was performed using proteins exhibiting a median absolute deviation (MAD) > 1 using Pearson correlations in Clustvis (version 1.2.0) in R (version 3.6.2). Co-quantified transcripts and proteins with a MAD > 0.5 were prioritized for unsupervised hierarchical clustering. Differential analysis of proteins and/or transcripts was performed using limma (version 3.8, [19]). RPPA abundances were log_2_-transformed and target-wise median centered. RPPA abundances below the limit of detection were converted to a small non-zero value (e^-10) prior to normalization. The variance between sampling levels for selected RPPA targets not quantified by MS which represent biomarkers relevant to ongoing clinical trials enrolling USC or other endometrial cancer patients was calculated from the log_2_-transformed target-wise median centered data. The significance of variance differences between LMD collection types were calculated by Mann Whitney U test. Only proteins in the MS dataset passing a limma adjusted p < 0.05 and exhibiting the same pattern of expression across all 9 patients were included in downstream analyses, including for comparison with proteins passing these criteria in the HGSOC study described previously [13], and association with known functional pathways using Ingenuity Pathway Analysis (IPA; Qiagen). The IPA-identified lists of associated drug targets were cross-referenced against Table 1 from Sun et al. [20] for identification of FDA-approved drug targets. Protein-level and RPM-level data were processed in xCell (version 1.1.0; [21]) for cell type enrichment analysis. Relative protein abundances or cell type signature scores were plotted using ggplot2 (version 3.2.1) [22]. Single-sample gene set enrichment analysis (ssGSEA) [23] was performed using the protein-level data using the GSVA [24] package (version 1.34.0) in R (version 3.6.0). Signatures used for ssGSEA were generated by incorporating differential expression analysis with support vector machine recursive feature elimination (RFE) on quantitative proteomic data from a linearly diluted series of in situ-derived HGSOC ET, ES, and immune cells [25]. The ssGSEA clustermap and boxplots were plotted in Python (version 3.9.16) with seaborn (version 0.11.2), matplotlib (version 3.7.0) [26], and stat annotations (version 0.5.0) [27]. Patient-specific dendrograms were generated using ggtree (version 2.0.1) and ape (version 5.3) using proteins with MAD > 1. Pairwise Spearman correlations were calculated using all protein-level abundances (i.e., no MAD cutoff) between all sampling levels per case; protein–protein correlations (excluding the Spearman R = 1 correlations of each level to itself) were plotted in a ridgeline plot using ggplot2 (version 3.4.1) [22]. Heatmaps depicting the Spearman correlations between co-quantified protein (LC–MS/MS) and transcript samples in Fig. 4 and between co-quantified protein (LC–MS/MS) and RPPA samples in Additional file 3: Figure S3 were generated using the Morpheus software from the Broad Institute (https://software.broadinstitute.org/morpheus/; version 1.0–1). Spearman correlations were calculated in MedCalc (version 20.109) using all proteins significantly co-altered between ET and ES (ie., from Additional file 8: Tables S16 and S18; n = 455 proteins total) with those co-quantified (n = 442 proteins from their 7908 proteins total) in BT collections from n = 9 USC tumors reported by CPTAC [28] which had tumor purity values calculated through analysis of methylation data [29] reported. Quantitative abundances for these 442 proteins were directly compared to the methylation-derived metrics of tumor purity (Purity_Cancer), as reported in Additional file 8: Table S1 from Dou Y et al. [28]. Stroma scores for the global proteome data from the CPTAC dataset were calculated using ProteoMixture [25] and directly compared to the methylation-derived stroma purity metrics (Purity_Stroma) from the CPTAC dataset [28].

## Results

### Multi-omic analysis of LMD enriched cellular subpopulations from USC tumor specimens

**Fig. 1:**
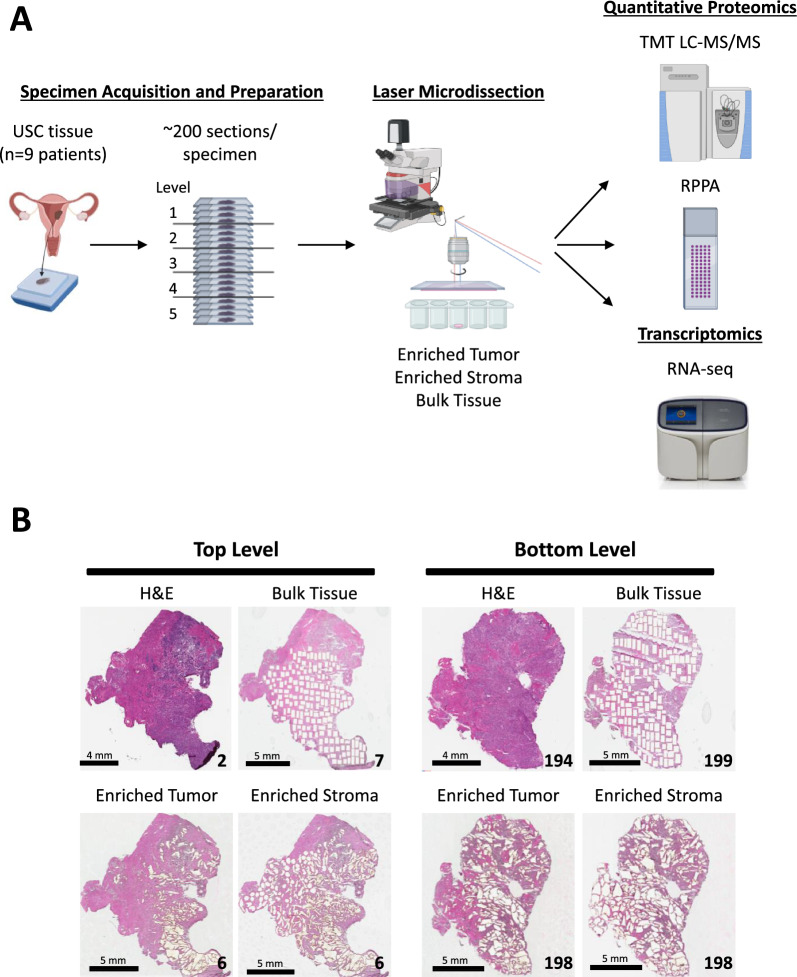
Study workflow. **A** Illustration of histological tissue specimen and LMD enriched sample preparation followed by quantitative proteomic [high-resolution liquid chromatography-tandem mass spectrometry (TMT LC–MS/MS) and reverse phase protein microarray (RPPA)] and transcriptomic (RNA-seq) analyses. Frozen tissue specimens from 9 USC patients were sectioned into ~ 200 consecutive thin tissue sections which were divided into 5 evenly distributed sampling levels (quintiles). Tissue sections within each level were laser microdissected for harvest of ET, ES, and BT to support each downstream analytical workflow. **B** Representative pre- and post-LMD micrographs from the top and bottom levels of tissue from case 343WC. The number in the bottom right corner of each micrograph indicates the section number. The scale bar in the bottom left corner of each micrograph indicates a length of 4 or 5 mm, as specified

Nine fresh-frozen USC patient tumor tissue specimens (Additional file 8: Table S1) were consecutively sectioned into ~ 200 tissue thin sections for subsequent LMD enrichment of tumor and stroma cell subpopulations for proteomic analysis. LMD enriched samples from 343VY were further generated for transcriptomic analysis. Representative cover-slipped hematoxylin and eosin (H&E)-stained sections interspersed throughout the depth of each block were reviewed by a board-certified pathologist (UNMR) to confirm histologic characteristics and tumor cellularity, which ranged from 15 to 99% (Additional file 8: Table S1). LMD enriched harvests of tumor epithelium (ET) or stromal cells (ES), as well as whole “bulk” tissue (BT) collections representing all tissue material from a single section, were obtained from five equally sized and spatially distinct regions of tissue (“levels”) from alternating sections throughout the specimen blocks for proteomic and/or transcriptomic analysis (Fig. 1A; Additional file 8: Table S2). Representative images were collected before and after LMD for quality control (Fig. 1B).

Quantitative proteomic (LC–MS/MS and RPPA) and transcriptomic (RNA-seq; patient 343VY only) analyses were performed using LMD enriched samples. LC–MS/MS analysis quantified an average of 9548 ± 450 proteins within each patient-specific TMT plex for a total of 6,503 proteins co-quantified across all nine patients (Additional file 8: Table S3). A total of 15,558 transcripts were quantified from 343VY (Additional file 8: Table S4). RPPA analysis was performed using 281 antibodies [13] to assess the expression of native and/or post-translationally modified proteins (Additional file 8: Table S5).

The global protein abundance matrix (Additional file 8: Table S3) from these nine patients can be accessed at our Heterogeneity Analysis Portal (https://lmdomics.org/), which we previously developed as a community resource for interrogation of a spatially resolved and LMD enriched sample series generated and molecularly profiled using similar methods from high-grade serous ovarian carcinoma (HGSOC) patient tumors [13].

### ET and ES collections exhibit unique molecular profiles of regional molecular heterogeneity

**Fig. 2:**
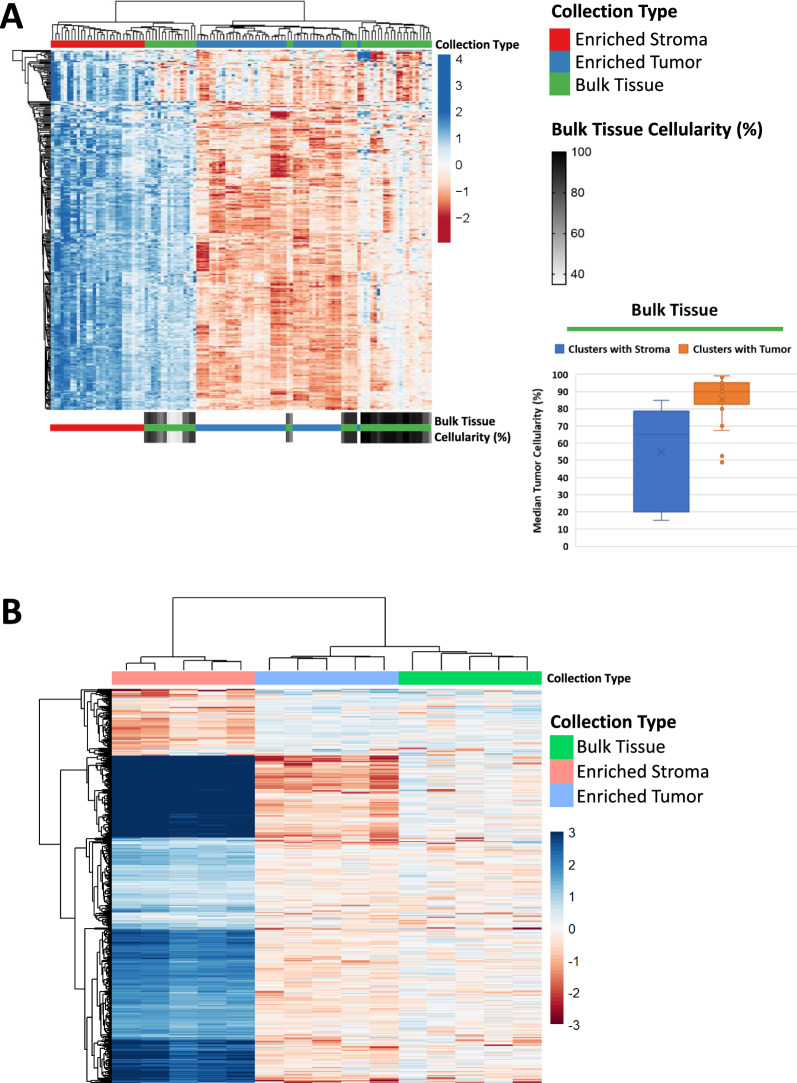
Unsupervised hierarchical cluster analysis of differentially abundant proteins and transcripts. **A** 351 variably abundant proteins (MAD > 1) and **B** 464 variably abundant transcripts (MAD > 0.5) co-quantified in both the RNA-seq and mass spectrometry datasets. **A** Protein abundances are represented across 118 samples derived from n = 9 patients consisting of ET (n = 44 total; 4–5 levels/patient), ES (n = 29 total; 2–5 levels/patient), and BT (n = 45 total; 5 levels/patient). The color gradient inset below the heatmap depicts median tumor purity estimates per level, determined by manual pathology review for each of the BT collections

Unsupervised hierarchical cluster analysis of 351 variably abundant proteins (median absolute deviation (MAD) > 1) revealed two predominant branches with independent clustering of the ET samples from ES (Fig. 2A). The association of BT with either ET or ES was related to tumor “purity”, as previously described [13]. Specifically, BT from specimens with overall higher or lower median tumor purity clustered with ET or ES, respectively. This result was further recapitulated in transcriptome data from 343VY, which showed similar, independent clustering of ET and ES samples (Fig. 2B). BT samples expectedly clustered with ET as the median tumor cellularity for each of the 5 levels from this case was ≥ 97% (Additional file 8: Table S1).

**Fig. 3:**
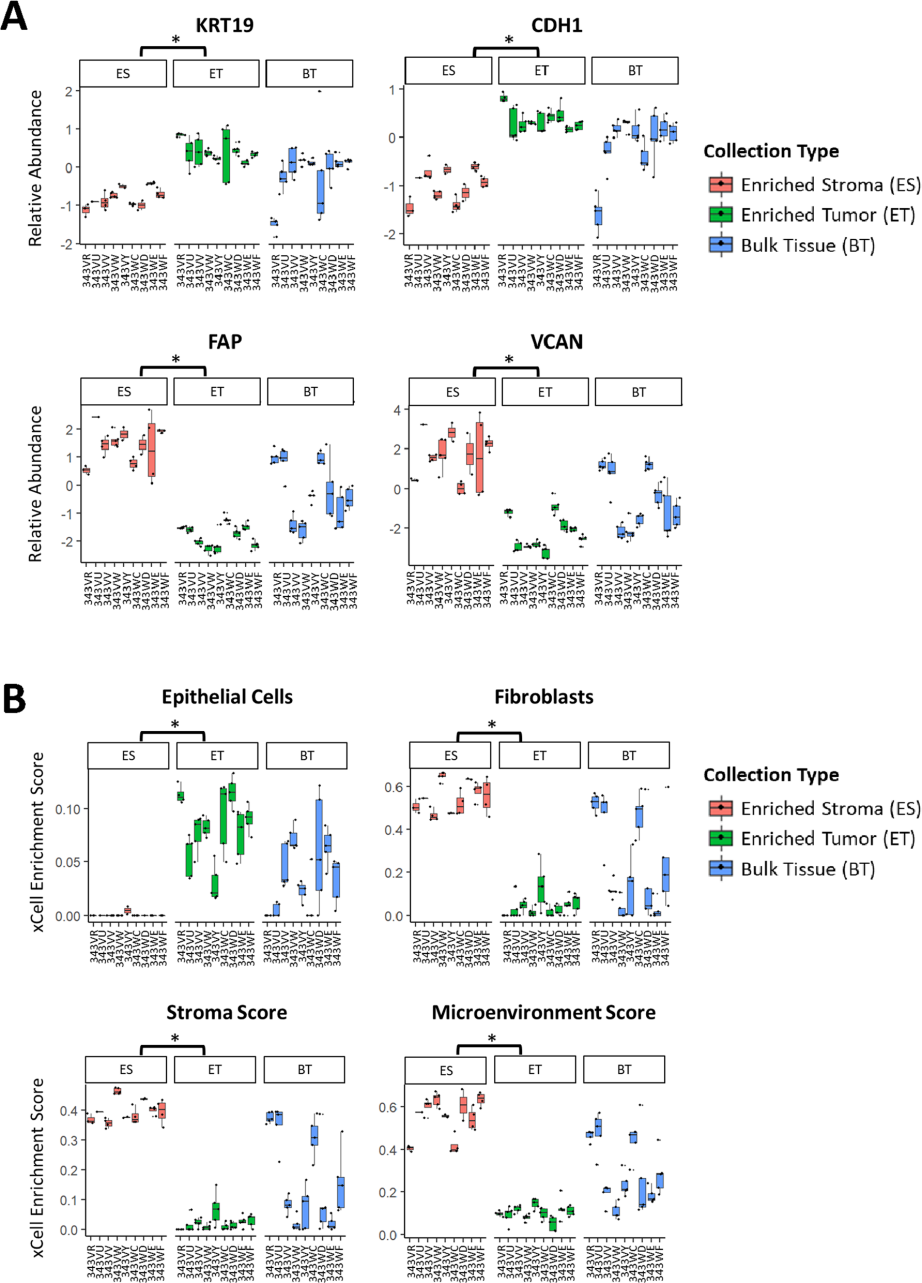
Protein abundance of cell type-specific markers and cellular admixture analyses (xCell [21]). **A** Boxplots depicting relative protein abundances of classical tumor (KRT19 and CDH1) and stroma (FAP and VCAN) markers. **B** Cell type signature scores as determined by xCell. Wilcox p-values with (*) indicate statistically significant differential expression (p < 0.0001) between ET and ES

Proteomic abundances of epithelial and stromal markers were examined for each collection type (Fig. 3A). The epithelial markers Keratin Type I Cytoskeletal 19 (KRT19) and Cadherin 1 (CDH1) were significantly elevated in ET relative to ES (Wilcox p < 0.0001). Conversely, stromal markers Fibroblast Activation Protein Alpha (FAP), and Versican (VCAN) were significantly elevated in the ES relative to ET (Wilcox p < 0.0001). Intermediate abundances in BT correlated with the relative proportions of tumor and stroma within the TME. Cell type enrichment scores (xCell [21]) using transcriptomic data from 343VY (Additional file 8: Table S6) and proteomic data from all nine cases (Additional file 8: Table S7) demonstrated enrichment of epithelial cell scores in ET, and enrichment of cell type signature scores for fibroblasts, stroma, and microenvironment in ES (Wilcox p < 0.0001; Fig. 3B), as previously described [13].

We also explored a single sample gene set enrichment analysis (ssGSEA) classifier enabling prediction of tumor, stroma, and immune cell admixture optimized for proteomic data (ProteoMixture [25]) for characterizing the ET, ES, and BT samples (Additional file 8: Table S8). BT samples with high tumor cellularity and ET had higher ssGSEA “tumor” scores, versus BT samples with low tumor cellularity and ES had higher ssGSEA “stroma” scores. The ssGSEA “immune” scores were more variable across sample types, though generally trended higher in ES and lower tumor cellularity BT samples (Additional file 1: Figure S1).

Serum levels of CA-125, a cleaved extracellular domain of the MUC16 protein, are used clinically as a biomarker for monitoring USC progression and regression. Similar to our findings in HGSOC [13], MUC16 in the present study was significantly elevated in ET relative to ES of USC patient specimens (Wilcox p < 0.0001; Additional file 2: Figure S2). We previously demonstrated that proteins possessing a signal peptide sequence and/or characterized as “extracellular” by Gene Ontology were more variably abundant than proteins without these characteristics [13]. Examination of 5 cases in the present study which had 4–5 sampling levels for both ET and ES revealed a significantly higher variance of proteins containing a signal peptide sequence and/or categorized as extracellular between sampling levels relative to proteins lacking these features (p < 0.0001; Additional file 8: Table S9).

Integrated analysis of the LC–MS/MS and RNA-seq data from 343VY revealed 6019 co-quantified protein:transcript pairs in both the proteomic and transcriptomic datasets. The protein and transcript abundances in LMD enriched samples were more strongly correlated (Spearman correlations ranging from 0.12 to 0.36 in ET, and 0.46 to 0.54 in ES; Additional file 3: Figure S3A, B; Additional file 8: Table S10) than those in BT (Spearman correlations ranging from − 0.05 to 0.06; Additional file 3: Figure S3C; Additional file 8: Table S10).

### Orthogonal proteomic analyses reveal intratumor heterogeneity in therapeutic drug targets

**Fig. 4:**
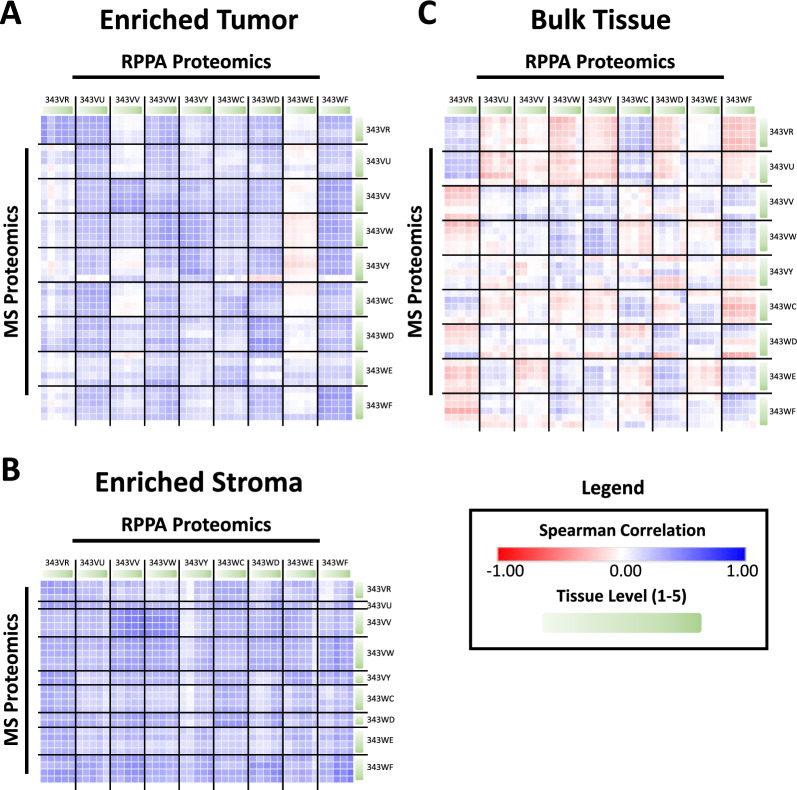
Correlation matrices of samples using proteins co-quantified by LC–MS/MS and RPPA. Spearman correlation analysis of samples using 160 proteins that were co-measured by TMT LC–MS/MS and RPPA for **A** ET, **B** ES, and **C** BT

RPPA was used to quantify the abundances of 281 native and/or post-translationally modified proteins (Additional file 8: Table S5) as described previously [13] for two important reasons: [1] as an orthogonal immunoassay-based approach cognate with our MS-based profiling efforts to characterize the heterogeneity in the phosphoproteome using two separate complementary proteomic-based methods, and [2] to leverage the strength of content provided by the RPPA platform as a tool that can generate semi-quantitative information about the expression and activation (ie, phosphorylation) of key protein drug targets and signaling pathway proteins. These total and/or phosphoproteins represent key “actionable” drug targets measured by RPPA from the LMD tissue, which may be below the detection limits of MS analysis. Of the 160 proteins that were co-quantified by both MS and RPPA (from 142 antibodies, some of which recognized multiple isoforms and/or phosphorylated epitopes of a protein, thus mapping to multiple Uniprot accessions), the strongest correlations were between ET and ES samples from the same patient, with intra-patient Spearman correlations between -0.03 to 0.44 (median = 0.31) and 0.07 to 0.54 (median = 0.31), respectively (Fig. 4A, B; Additional file 8: Table S11). The BT collections were often poorly correlated both within (Spearman correlations of − 0.32 to 0.37; median = 0.10) and between (minimum Spearman correlation of − 0.36) patients (Fig. 4C; Additional file 8: Table S11).

A total of 119 proteins (corresponding to 139 unique antibodies) were exclusively assayed by RPPA, while not concurrently quantified by MS. Notable targets among these which have demonstrated therapeutic significance when targeted in recent endometrial cancer clinical trials (Additional file 4: Figure S4) include programmed cell death protein 1 and programmed death-ligand 1 (PD-1/PD-L1) [10–12]. The clinical benefit of selinexor, a selective inhibitor of exportin 1 (XPO1), as a maintenance therapy was recently demonstrated in endometrial cancer patients with wild-type tumor protein 53 (TP53) [30]. TP53 and phosphorylated (p)TP53^S15^ were assayed by RPPA and not quantified by MS. Checkpoint kinase 1 (CHK1^S345^), selectively inhibited by prexasertib (ACR-368), was exclusively quantified by RPPA and has demonstrated early promising results as a therapeutic target in platinum-resistant endometrial cancer (NCT05548296). While human epidermal growth factor receptor 2/3 (HER2/3^Total^) were quantified by MS, RPPA further quantified several phosphorylated epitopes (pHER2^Y1248^, pHER2^Y877^, pHER3^Y1289^) with relevance to clinical trials investigating HER2-targeting agents in patients with USC and/or other endometrial cancer subtypes (NCT05256225, NCT04704661, NCT04486352). Among these notable markers, the variance between sampling levels was significantly improved by LMD enrichment of tumor for CHK1^S345^, HER3, and HER3^Y1289^ relative to the BT harvests (p < 0.05).

The identities of the proteins quantified in the MS dataset and which were most positively or negatively correlated (all p < 0.01) with their native and/or modified forms co-quantified in the RPPA dataset were examined in each LMD collection type (Additional file 8: Table S12). Intercellular adhesion molecule 1 (ICAM1), lamin A (LMNA; cleaved D230), annexin II (ANXA2), focal adhesion kinase I (PTK2^Y576/577^), and annexin I (ANXA1) were the most highly correlated proteins in ET, whereas syndecan I (SDC1), mitogen activated protein kinase 8 (MAPK8^T183/Y185^), proto-oncogene tyrosine-protein kinase Src (SRC^Y416^), tyrosine-protein kinase JAK1 (JAK1^Y1022/1023^), and protein kinase C beta type (PRKCB^S600^) were the most negatively correlated. ANXA1/2 were similarly highly correlated in ES, in addition to diacylglycerol kinase alpha (DGKA), phosphatidylinositol 3,4,5-trisphosphate 5-phosphatase 1 (INPP5D^Y1020^), and HLA class II histocompatibility antigen DR alpha chain (HLA-DRA). Proteins negatively correlated between MS and RPPA in ES included myristoylated alanine-rich C-kinase substrate (MARCKS^S152/156^), beta arrestin-1 (ARRB1^S412^), cyclin B1 (CCNB1), dual specificity mitogen-activated protein kinase kinase 1 (MAP2K1^S298^), and glucocorticoid receptor (NR3C1^S211^). While ANXA1/2 were highly correlated in both ET and ES, they were not correlated (p > 0.05) in BT samples. The most correlated proteins in BT included integrin-linked protein kinase 1 (ILK), phosphatidylinositol 3,4,5-trisphosphate 3-phosphatase and dual-specificity protein phosphatase PTEN (PTEN^S380^), cyclin-dependent kinase 2 (CDK2), heat shock protein HSP 90-beta (HSP90AB1), and poly [ADP-ribose] polymerase 1 (PARP1). The most negatively correlated proteins in BT were cytosolic phospholipase A2 (PLA2G4A^S505^), cAMP-dependent protein kinase catalytic subunit beta (PRKACB^T197^), SDC1, SRC^Y416^, and LMNA (cleaved D230).

### Sampling levels within a tumor specimen display variable degrees of proteomic relatedness

**Fig. 5:**
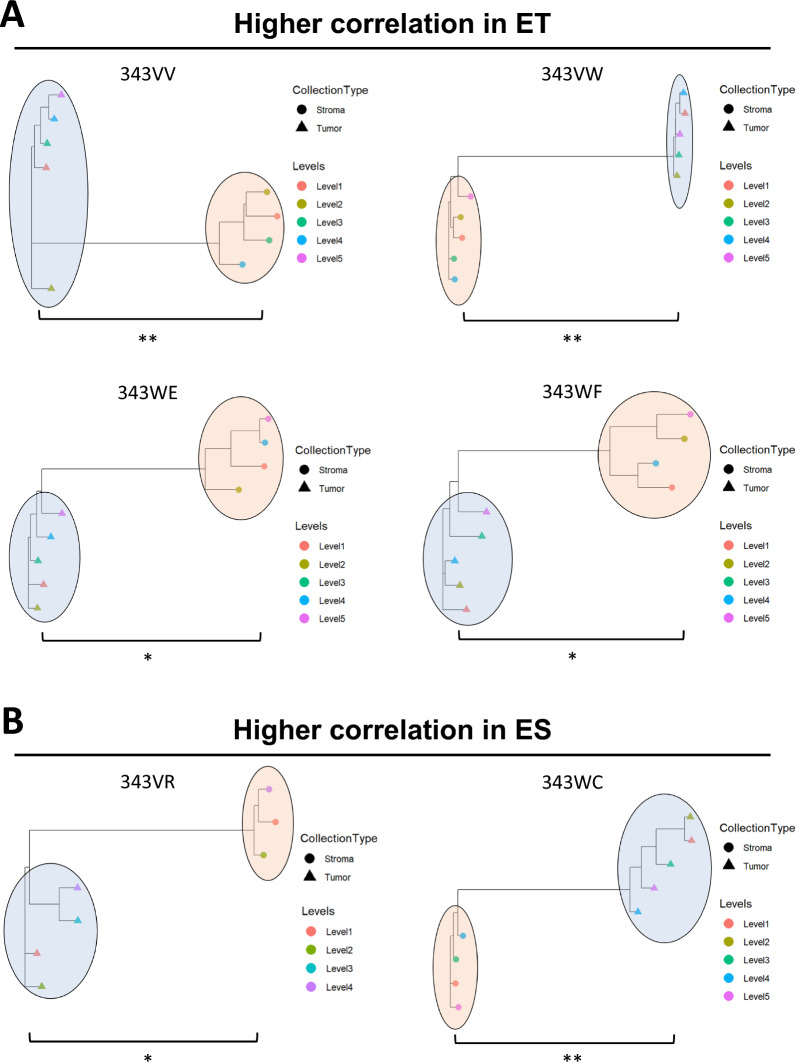
Patient-specific phylogenetic analyses. Dendrograms were constructed using Spearman correlations based on differentially expressed proteins between ET and ES. Spearman correlations were calculated within ET and ES for cases 343VR, 343VV, 343VW, 343WC, 343WE, and 343WF using the abundances of 106, 145, 1,061, 263, 175, and 278 proteins with MAD > 1, respectively. Statistically significant differences between LMD collection types are shown with (*) for p < 0.05 and (**) for p < 0.01. **A** Cases with higher Spearman correlations in ET. **B** Cases with higher Spearman correlations in ES

The heterogeneity between collection levels within and between LMD enriched sample types was examined using a phylogenetic approach, as previously described [13], using six cases for which there were ≥ 3 LMD collection levels available from ET and ES samples (Fig. 5; Additional file 8: Table S13). The degree of heterogeneity measured between ET and ES significantly differed (p < 0.05 or p < 0.01, as noted) within each of the 6 cases, however there was no consistent trend across all patients of whether the degree of protein expression variability between collection levels was greater in ET or ES. ET had higher correlation between collection levels (i.e., had less heterogeneity in protein expression patterns) in 4 cases (Fig. 5A), while the ES from 2 cases were better correlated (Fig. 5B). The two cases that had higher correlation between ES samples (and correspondingly more heterogeneity between ET samples) had lower overall median tumor cellularity per sampling level (Additional file 8: Table S1).

We further examined pairwise correlations between BT samples from each case using the proteomic abundances quantified by LC–MS/MS analysis (Additional file 5: Figure S5). The median intra-patient, inter-level Spearman correlation of BT samples was 0.54 (range − 0.50 to 0.84; Additional file 8: Table S14). The pairwise comparisons of BT samples from several levels were negatively correlated in cases 343VY and 343WD, although these specimens had the highest tumor cellularity (range 97–99% and 60–95%, respectively; Additional file 8: Table S1). Conversely, case 343VR had the lowest tumor cellularity (range 15–20%; Additional file 8: Table S1) and the highest correlations between BT sampling levels (Additional file 8: Table S14). Collectively, these trends could reflect clonal heterogeneity present in the tumor, that is not also present in the stroma, thus warranting future investigation and highlighting the need for intratumor multiregion sampling.

### Comparative analysis of cell type specific biomarkers in gynecologic serous carcinomas

**Fig. 6:**
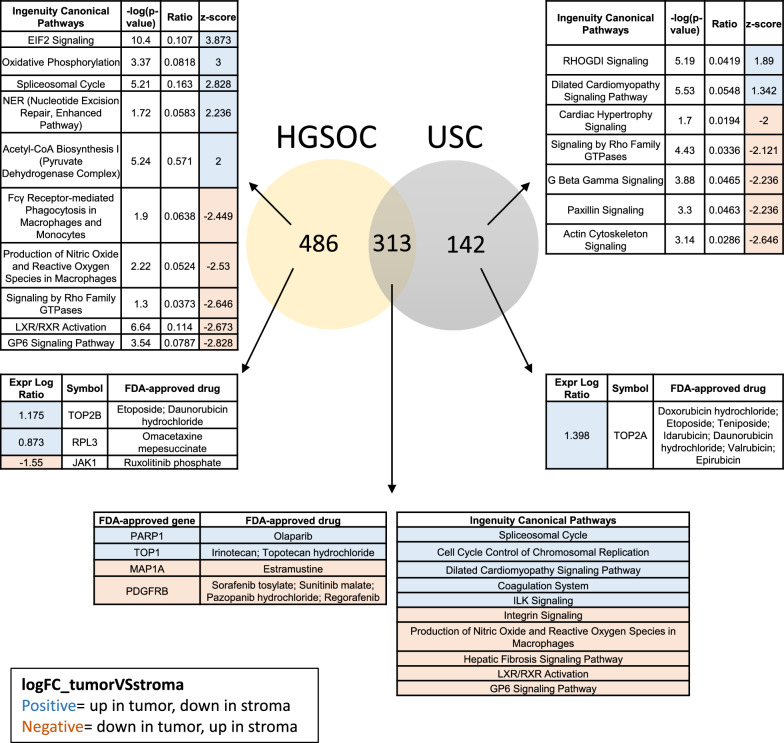
Disease-specific alterations in USC versus HGSOC tissue specimens. Comparative analysis was performed using differentially expressed proteins in ET versus ES from USC and HGSOC specimens. Proteins passing limma adjusted p < 0.05 with the same pattern of enrichment (i.e., positive or negative LogFC values) across all patients within the respective USC (n = 455 proteins from Additional file 8: Table S3) and HGSOC (n = 796 proteins from Hunt et al. Additional file 8: Table S7 [13]) datasets were prioritized for comparative analyses between serous carcinoma types. The top 5 significantly altered canonical pathways identified by Ingenuity Pathway Analysis (IPA) are highlighted. Drug targets identified by IPA were further crossed with those that are FDA-approved [20]. Canonical pathways and drug targets with positive z-scores (highlighted blue) are elevated in ET, while those with negative z-scores (highlighted red) are elevated in ES

To examine the proteomic similarities between two serous subtype gynecologic carcinomas, USC and HGSOC, we compared the significantly altered proteins between ET and ES from the present USC study with those in our previous HGSOC dataset [13]. A total of 313 proteins were commonly significantly altered (LIMMA adj. p < 0.05) and exhibited the same abundance trends between ET and ES in both datasets (HGSOC LogFC values in Additional file 8: Table S15; USC LogFC values in Additional file 8: Table S16). There were 483 (Additional file 8: Table S17) and 142 (Additional file 8: Table S18) significantly altered proteins unique to the HGSOC and USC datasets, respectively (Fig. 6).

We prioritized investigation of FDA-approved drug targets [20] and the top 5 IPA canonical pathways (by z-score; Fig. 6). The complete lists of all drug targets and canonical pathways analyzed (p < 0.05) are reported in Additional file 8: Table S19. Rho GDI pathway signaling was uniquely elevated in ET of USC specimens, based on the differential expression of 9 pathway-related proteins. Rho GDP-dissociation inhibitors 1 and 2 (ARHGDIA and ARHGDIB) were not included in this comparative analysis as they exhibited discordant abundance trends between patients, but both proteins were elevated overall in ES from both USC and HGSOC (limma p < 0.001). Conversely, pathway activity for RhoA signaling was lower in USC ET. Notably however, the abundance of the RhoA protein itself did not vary between collection types (limma p = 0.62, USC). The top 5 pathways that were uniquely elevated in ET from HGSOC patients included EIF2 signaling, oxidative phosphorylation, splicesosomal cycle, nucleotide excision repair, and acetyl-CoA biosynthesis.

Pathways commonly enriched in ET from both USC and HGSOC include the splicesosomal cycle (using proteins significantly altered in both HGSOC and USC), cell cycle control of chromosomal replication, and dilated cardiomyopathy signaling. Semaphorin signaling was enriched in HGSOC ET while comparatively also enriched in USC ES, through coverage of different sets of pathway-related proteins. The coagulation system and ILK signaling were additionally commonly enriched in ET of both serous cancer types.

A 4-gene (*KRT23*, *CXCL1*, *SOX9*, and *ABCA10*) prognostic signature that stratifies USC patients by risk and overall survival (OS) was recently reported [31]. CXCL1 and ABCA10 were not measured in our proteomic dataset. SOX9 was significantly higher in ET (Wilcox p < 0.001), whereas KRT23 was elevated in ES (Wilcox p < 0.05) from most (notably not all) cases (Additional file 6: Figure S6).

Stromal expression of nicotinamide N-methyltransferase (NNMT) is correlated with disease progression and metastasis in high grade endometrial cancers (serous and others), in which elevated levels of stromal NNMT in the primary and metastatic tumors are associated with poor overall survival [32]. We identified NNMT as significantly elevated in our ES samples (Wilcox p < 0.0001; Additional file 7: Figure S7).

**Fig. 7:**
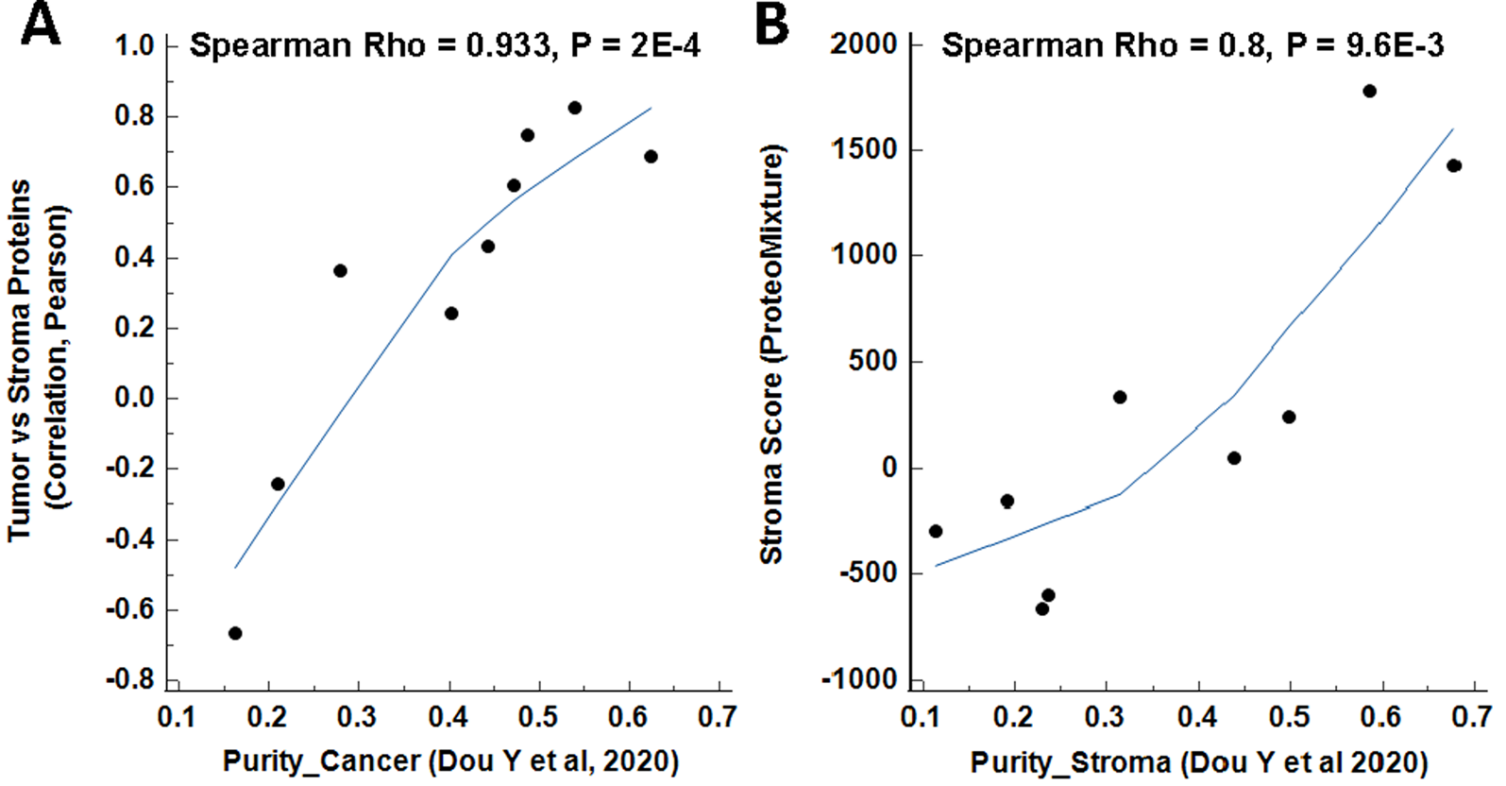
Comparison of proteins altered between ET and ES in USC and stromal admixture scores with proteomic data generated from BT collections of USC tumors reported by Dou Y et al. 2020 [28]. **A** Proteins significantly co-altered (limma adj. p < 0.05) between ET and ES for n = 9 USC tumors (from Additional file 8: Tables S16 & S18; n = 455 proteins total) were correlated with global proteome data from bulk tissues collections for n = 9 USC tumors reported by Dou Y et al. Among 7908 total proteins quantified in all samples by Dou Y et al., 442 proteins were co-quantified in our cohort with tumor and stroma alterations. Correlation of the quantitative abundances for these 442 proteins were directly compared to metrics of tumor purity derived from analysis of methylation data (Purity_Cancer) as reported in Dou Y et al. **B** Stroma scores were calculated for global proteome data reported by Dou Y et al. using ProteoMixture [25] and directly compared to stroma purity metrics derived from methylation data (Purity_Stroma) reported by Dou Y et al.

We further correlated the expression of conserved proteomic alterations between ET and ES from our USC cohort with the subset of USC tumors included within the Clinical Proteomic Tumor Analysis Consortium (CPTAC) uterine corpus endometrial carcinoma (UCEC) dataset [28], prioritizing nine CPTAC USC tumors which had methylation-derived tumor and stroma purity metrics reported (Fig. 7A). Of the 455 total significant protein alterations (collectively from Additional file 8: Tables S16 and S18) between in ET versus ES in our dataset, 422 were co-quantified in the CPTAC tumors and were highly correlated (Spearman Rho = 0.93, p < 0.001). Analysis of the CPTAC proteomic data using ProteoMixture [25] and further comparison of the ProteoMixture stromal scores with the inferred stromal purity scores revealed high correlation (Spearman Rho = 0.8, p < 0.01) of CPTAC purity values with the ProteoMixture stromal score (Fig. 7B). Collectively, these data underscore that tumors from the CPTAC dataset included several of low purity (< 50% tumor cellularity) without consideration of cell type deconvolution during sample preparation.

**Fig. 8:**
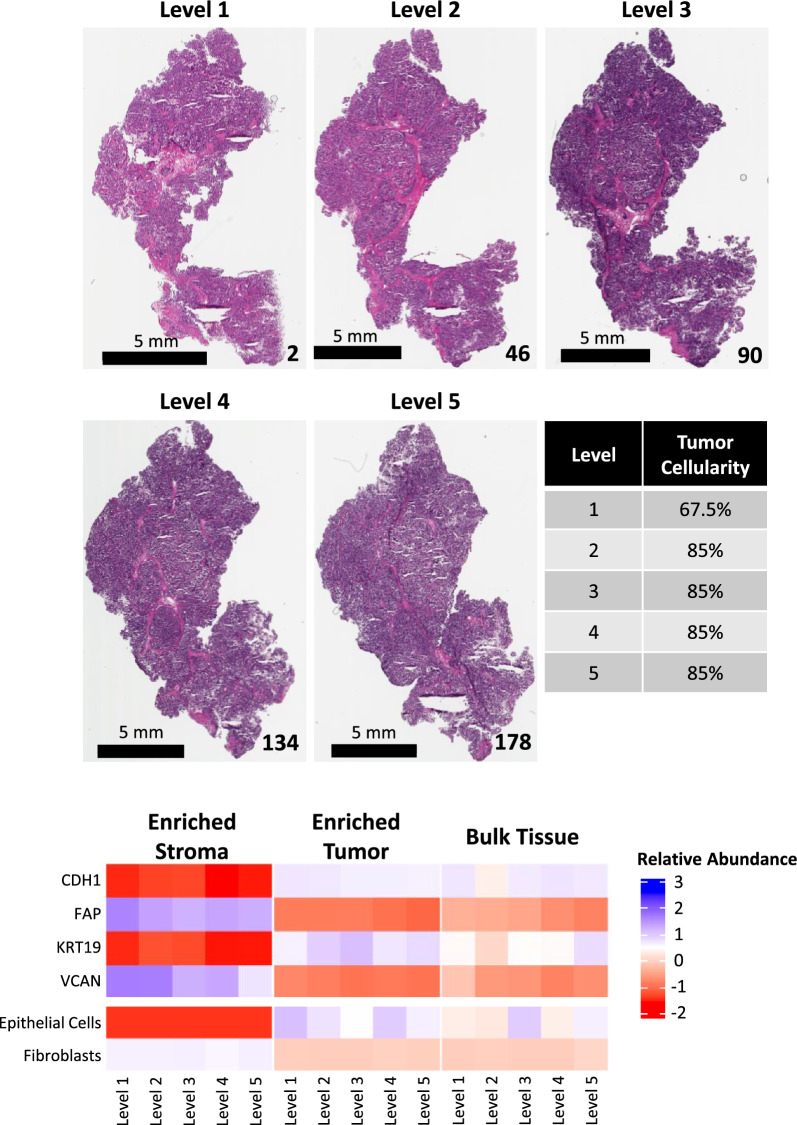
Representative case (343VW) depicting variable molecular expression by LMD sampling level. Representative micrographs of H&E-stained tissue sections mounted on glass slides from the top of each sampling level. The tissue section number is notated in the bottom right corner. The scale bar in the bottom left corner of each micrograph indicates a length of 5 mm. The median tumor cellularity (%), as determined by manual pathology review, per level with relative standard deviation (%CV) is shown (middle table). The proteomic abundances of representative tumor/epithelial (CDH1 and KRT19) and stroma (FAP and VCAN) markers, and xCell cell type enrichment scores for epithelial cells and fibroblasts [21] are shown in the heatmap

Finally, case 343VW was selected as a representative to highlight the extent of heterogeneity observable within a single USC tumor specimen because the histological composition was highly consistent throughout the depth of the specimen block (Fig. 8, Additional file 8: Table S1). Proteomic expression of epithelial (CDH1 and KRT19) and stromal (FAP and VCAN) markers were consistent in ET and ES specimens, respectively. Notably, the abundance of these protein markers in BT specimens was more variable as shown by the mixed patterns of CDH1 and KRT19 expression, minimal representation of known stromal markers, and variable cell type classification scores (admixture scores for epithelial cells and fibroblasts) [21] across sampling levels.

## Discussion

Uterine cancer, of which USC represents a particularly aggressive histotype, is one of the few malignancies with increasing incidence and mortality rates [1], warranting critical need for improved disease characterization and identification of targetable biomarkers. The contributions of stromal-derived disease contributors are increasingly being recognized for HGSOC [33–35], pancreatic ductal adenocarcinoma [36], and breast cancer [37]. Indeed, examination of proteomic abundances for genes relating to a USC prognostic signature [31] in the present study revealed that while SOX9 was generally seen to be elevated in the epithelium (Wilcox p < 0.001), KRT23 was elevated in the stroma from most cases (Wilcox p < 0.05). NNMT is also a biomarker of USC disease aggressiveness where it has been shown to be elevated in the stroma of primary high grade endometrial tumors (relative to benign endometrial tissue), and further elevated in metastases [32]. We consistently observed significantly elevated NNMT expression in the stroma (Wilcox p < 0.0001). These results collectively emphasize that decoupling of cellular subpopulations within the TME through LMD, single cell proteomics and/or sequencing, or in situ molecular analysis is necessary for identification and quantification of disease markers of tumor epithelium and stromal origin [13, 38–40]. Unique proteomic and transcriptomic profiles are present in both epithelium and stroma, each with locoregional heterogeneity relating to spatial distribution throughout the cancer TME.

Serous cancers of uterine and ovarian primary origins have several morphological [41], chromosomal [42], genomic and/or proteomic similarities [42, 43] that complicate clinical distinction and diagnosis. Both USC and HGSOC are typified by chromosomal instability, somatic copy number alteration (SCNA), and frequent mutations in *TP53* and other genes. Specifically, a recent analysis using TCGA data co-identified mutations in *MUC16*, *FLG*, and *AHNAK* in both serous cancers, though the incidences were < 25% [42]. Historically, clinical management of both USC and HGSOC has largely consisted of platinum and paclitaxel-based chemotherapy regimens, but response rates to biologic therapies (i.e. immune checkpoint inhibitors, PARP inhibitors, etc.) may differ due to differences in targetable alterations as well as adaptive chemoresistant pathways [44]. Comparative analysis of significantly altered proteins between ET and ES in the current USC dataset with those from a previous HGSOC dataset [13] revealed several pathways that were commonly enriched in both serous cancer types, as well as several that were uniquely measured in only one of the respective datasets. An improved understanding of both commonalities as well as differences between these serous tumors provides opportunities for treating some gynecologic cancers on the basis of histology whereas others may require different therapeutic approaches based on both histology and the organ of origin (i.e. tube/peritoneum/ovary and endometrium).

Rho guanine nucleotide dissociation inhibitors (GDIs) and their interactions with Rho family proteins have known involvement in several malignancies, though their specific activity and enrichment differ between cancer types [45]. GDIs are involved in the regulation of Rho activity through inhibition of GTP binding [46] and/or extraction of membrane-bound Rho GTPases for storage of the inactivated protein in the cytosol, while protecting the cytosolic Rho GTPases from proteolytic degradation [47, 48]. In our study, we observed that Rho GDI signaling was significantly enriched in USC ET, while RhoA signaling was conversely enriched in USC ES. Activation of Rho GDI and RhoA signaling were not differentially localized in the HGSOC dataset [13], despite the known elevation of transcriptomic and proteomic abundances of RhoA, RhoC [49], and RhoGDI2 [50] in invasive HGSOC.

Several pathways typified by expression of MYL6, MYL9, and RRAS were uniquely differentially enriched in USC only, in which they were elevated in ES. MYL6 and MYL9 are myosin light chain family polypeptides specific for non-muscle myosin II [51], which are not normally expressed in endometrial stroma [52] and are involved in semaphorin pathway signaling [53]. Semaphorin signaling was elevated in USC ES and has previously been implicated as a targetable pathway for endometrial cancer treatment. Specifically, upregulation of SEMA3B and SEMA3F following progesterone (P4) and 1,25-dihydroxyvitamin D3 treatment increases caspase-3 activity, thereby inhibiting the growth of cancer cells [54]. Comparatively, proteins relating to semaphorin signaling were also differentially localized in HGSOC, though the pathway was instead elevated in HGSOC ET and was identified through the quantification of a separate set of pathway-related proteins.

Through integrated interrogation of several native and post-translationally modified proteins via RPPA analysis and their co-quantified proteomic abundances in the MS data, we report the strength of correlation for known cancer-related proteins, including several in the drug target space, and the variability of these correlations within LMD enriched versus bulk tissue samples. ICAM1 was the most highly correlated protein in ET by RPPA and MS. ICAM1 overexpression is correlated with reduced recurrence-free and overall survival in HGSOC [55], and is a STAT1-associated gene with known correlation with disease metastasis in several cancers, including serous papillary endometrial cancer [56]. ANXA2, whose expression was highly correlated in both ET and ES, is predictive of recurrence in endometrial carcinoma [57]. Comparatively, ANXA2 abundances were poorly correlated in BT. Notably, the abundances of several proteins involved in mismatch repair (MLH1, MSH2, and MSH6) quantified by RPPA and MS were not correlated in any of the LMD enriched or bulk tissue collections (p > 0.05). Differential MMR proficiency has been demonstrated in several recent phase 3 clinical trials to impact the responsiveness to immune checkpoint inhibition administered in combination with chemotherapy [10, 12].We acknowledge that our analysis of specimens from nine USC patients represents a limitation regarding how accurately the heterogeneity observed here is generalizable to all USC cancer tissues. Our investigation draws significant strength from the numerous sampling levels that were harvested and analyzed within each patient specimen, which is not routinely performed in large-scale proteomic and/or multi-omic studies (including those from TCGA and CPTAC) for independent (decoupled) molecular analysis of ET and ES, and further from the integration of multi-omic analysis via quantitative proteomics (LC–MS/MS and RPPA), and transcriptomics (RNA-seq, for patient 343VY only). Future validation studies utilizing LMD enriched tissues from larger patient cohorts remains important. Lastly, we have compiled the data from this study and it can be accessed through our Heterogeneity Analysis Portal (https://lmdomics.org/), which represents a community resource to examine protein level heterogeneity throughout the TME in USC patient specimens.

## Conclusions

Extensive three-dimensional heterogeneity exists within the USC tumor tissue microenvironment, with disease-relevant biomarkers present in both the tumor and the stroma. These data underscore the critical need for upfront enrichment of cellular subpopulations from tissue specimens for spatial proteogenomic analysis.

### Supplementary Information

**Additional file 1: Figure S1.** ProteoMixture [25] ssGSEA scores of ET, ES, and BT samples. **A** Stacked heatmap depicting ProteoMixture ssGSEA scores for tumor, immune, and stroma in individual sampling levels from ET, ES, and BT harvests. **B** Boxplots of ssGSEA score enrichment by LMD collection type. Statistically significant differences between collections types are shown with (*) for p<0.01 and (****) for p<0.0001. *NS* not significant.

**Additional file 2: Figure S2.** Boxplot depicting relative protein abundance for MUC16. Asterisk (*) indicates significant difference between ES and ET (Wilcox p<0.0001).

**Additional file 3: Figure S3.**Protein-RNA Spearman Correlation Matrix for case 343VY. Spearman correlation analysis of 6,019 genes that were co-measured as proteins and corresponding transcripts in 343VY. Size and color of each circle reflects Spearman correlation.

**Additional file 4: Figure S4.** Variance between sampling levels for each LMD collection type of selected RPPA analytes not quantified by MS. Selected analytes represent biomarkers relevant to ongoing clinical trials enrolling patients with USC and/or other endometrial cancers.

**Additional file 5: Figure S5.** Ridgeline plot of pairwise Spearman correlations between BT harvests per case. The red-white-blue color scale represents Spearman correlations calculated from pairwise comparasions of each BT sampling level for the specified case. The greyscale color of the individual points (n=20 per case) represent the median tumor cellularity. The minimum and maximum values of median tumor cellularity per case are notated on the y-axis. The vertical height of peaks on the y-axis represent the density of the data points (correlations), scaled to 1.

**Additional file 6: Figure S6.** Boxplots depicting relative protein abundances for SOX9 and KRT23. Asterisks indicate significant difference between ES and ET. A single asterisk (*) represents Wilcox p<0.05; double asterisks (**) represent Wilcox p<0.0001.

**Additional file 7: Figure S7.** Boxplot depicting relative protein abundance for NNMT. Asterisk (*) indicates significant difference between ES and ET (Wilcox p<0.0001).

**Additional file 8: Table S1.** Clinical features and manual pathology assessment of tumor purity throughout the depths of USC patient specimen blocks. Representative H&E stained tissue sections on glass slides were examined at ~100 µm intervals by a board-certified pathologist for evaluation of the relative contributions (as percentages) of tumor cellularity, stroma, necrosis, normal ovarian epithelium, lymphocytes, and polymorphonuclear leukocytes (PMN) to the overall tissue composition. Multiple images per level/case were reviewed; the median estimates of tumor and stroma cellularity are reported with corresponding coefficient of variation (%CV) reported in parentheses. Abbreviations: NACT= neoadjuvant chemotherapy; NOS= not otherwise specified. **Table S2.** Depiction of study cohort. Numerical values indicate the number of LMD tissue sections that were used for each collection. Greyed boxes represent samples that were not collected or did not have sufficient yield of the target analyte for analysis. **Table S3.** Global protein matrix. Log_2_ transformed fold-change abundances of 6503 proteins co-quantified across all samples (n=118). **Table S4.** Transcriptome matrix for case 343VY. Log_2_-transformed normalized abundances of 15,558 RNA transcripts measured in case 343VY calculated relative to the average RPM abundance quantified across all samples for a given transcript. **Table S5.** Log_2_-transformed target-wise median centered RPPA abundances of protein and phosphoprotein targets in ET, ES, and BT samples. **Table S6.** Cell type enrichment scores using transcriptomic data for case 343VY in xCell (http://xcell.ucsf.edu/, [21]). **Table S7.** Cell type enrichment scores using proteomic data in xCell (http://xcell.ucsf.edu/, [21]). Table S8. ssGSEA scores calculated from “tumor”, “stroma”, and “immune” classifiers. **Table S9.** Median absolute deviation (MAD) of LMD enriched samples expressing or lacking signal peptide sequences and extracellular classification. P-values indicate the reliability of the presence or absence of a signal peptide or extracellular classification within the indicated LMD enriched tissue across all levels/case. **Table S10**. Spearman correlations for co-quantified proteins and transcripts from case 343VY. Spearman correlations were calculated using Log_2_ transformed fold-change abundances of 6,019 imputed proteins that were co-measured as transcripts. **Table S11.** Spearman correlations between samples using proteins co-quantified by MS and RPPA. **Table S12.** Spearman correlations for proteins co-quantified by MS and RPPA. **Table S13.** Pairwise Spearman correlations within and between ET and ES samples using proteins with MAD>1 for construction of patient-specific dendrograms. **Table S14.** Pairwise Spearman correlations between BT harvests using proteomic abundances. **Table S15.** LogFC values of proteins measured in HGSOC specimens which were commonly differentially expressed (limma adj. p<0.05) in ET and ES from USC specimens. LogFC protein abundances from Hunt *et al* Table S7 [13] which displayed the same pattern of expression and passed limma adj. p<0.05 across all patients were prioritized for comparative analysis with LMD enriched samples from USC specimens. The median LogFC values for 313 proteins co-altered from HGSOC samples, which were used as input for Ingenuity Pathway Analysis (IPA). Proteins reported in this table correspond to the HGSOC LogFC values for proteins in the center panel of the venn diagram in Fig. 6. **Table S16.** LogFC values of proteins measured in USC specimens which were commonly differentially expressed (limma adj. p<0.05) in ET and ES from HGSOC specimens. LogFC protein abundances from USC LMD enriched samples which displayed the same pattern of expression across all patients were prioritized for comparative analysis with LMD enriched samples from HGSOC specimens. The median LogFC values for 313 proteins co-altered from USC samples, which were used as input for Ingenuity Pathway Analysis (IPA). Proteins reported in this table correspond to the USC LogFC values for proteins in the center panel of the venn diagram in Fig. 6. **Table S17.** LogFC values of proteins measured in HGSOC specimens which were uniquely differentially expressed (limma adj. p<0.05) in ET and ES, which were not co-altered in LMD enriched samples from USC specimens. The 483 proteins reported in this table correspond to the HGSOC only LogFC values for proteins in the left panel of the venn diagram in Fig. 6. **Table S18.** LogFC values of proteins measured in USC specimens which were uniquely differentially expressed (limma adj. p<0.05) in ET and ES, which were not co-altered in LMD enriched samples from HGSOC specimens. The 142 proteins reported in this table correspond to the USC only LogFC values for proteins in the right panel of the venn diagram in Fig. 6. **Table S19.** Drug targets and canonical pathways identified by Ingenuity Pathway Analysis (IPA) significantly altered in ET and ES from USC and/or HGSOC specimens. Targets and pathways designated as “HGSOC only” correspond to the those identified using the 483 uniquely differentially expressed proteins between ET and ES from Additional file 8: Table S16 (Fig. 6, left panel of venn diagram). Targets and pathways designated as “USC only” correspond to the those identified using the 142 uniquely differentially expressed between ET and ES from Additional file 8: Table S17 (Fig. 6, right panel of venn diagram). Targets and pathways designated as “Overlap” correspond to those identified when using the HGSOC and USC (as specified) LogFC values of the 313 proteins in Additional file 8: Tables S14 and S15, respectively, as input.

## Data Availability

This study did not generate any unique reagents. The mass spectrometry proteomics data have been deposited to the ProteomeXchange Consortium via the PRIDE [58] partner repository with the dataset identifier PXD044197. The RNA-seq data will be provided upon request.

## Abbreviations

ANXA1: Annexin I
ANXA2: Annexin II
ARRB1: Beta arrestin-1
ARHGDIA: Rho GDP-dissociation inhibitor 1
ARHGDIB: Rho GDP-dissociation inhibitor 2
bRPLC: Basic reversed-phase liquid chromatography
BT: Bulk tissue
CCNB1: Cyclin B1
CDH1: Cadherin 1
CDK2: Cyclin-dependent kinase 2
CHK1: Checkpoint kinase 1
CPTAC: Clinical Proteomic Tumor Analysis Consortium
DGKA: Diacylglycerol kinase alpha
ES: Enriched stroma
ET: Enriched tumor
FAP: Fibroblast activation protein alpha
FDA: U.S. Food and Drug Administration
FDR: False-discovery rate
GDI: Guanine dissociation inhibitor
H&E: Hematoxylin and eosin
HCD: High-energy collisional dissociation
HER2/3: Human epidermal growth factor receptor 2/3
HGSOC: High grade serous ovarian carcinoma
HLA-DRA: HLA class II histocompatibility antigen DR alpha chain
HSP90AB1: Heat shock protein HSP 90-beta
ICAM1: Intercellular adhesion molecule 1
ILK: Integrin-linked protein kinase 1
INPP5D: Phosphatidylinositol 3,4,5-trisphosphate 5-phosphatase 1
IPA: Ingenuity Pathway Analysis
JAK1: Tyrosine-protein kinase JAK1
k-NN: K-nearest neighbor
KRT19: Keratin Type I Cytoskeletal 19
LC–MS/MS: Liquid chromatography tandem mass spectrometry
Limma: Linear models for microarray and RNA-seq data
LMD: Laser microdissection
LMNA: Lamin A
LogFC: Log_2_-transformed fold-change
MAD: Median absolute deviation
MAP2K1: Dual specificity mitogen-activated protein kinase 1
MAPK8: Mitogen activated protein kinase 8
MARKS: Myristoylated alanine-rich C-kinase substrate
NACT: Neoadjuvant chemotherapy
NNMT: Nicotinamide N-methyltransferase
NR3C1: Glucocorticoid receptor
NTC: No-template control
PARP1: Poly [ADP-ribose] polymerase 1
PD-1: Programmed cell death protein 1
PD-L1: Programmed death-ligand 1
PEN: Polyethylene naphthalate
PLA2G4A: Cytosolic phospholipase A2
PRKACB: CAMP-dependent protein kinase catalytic subunit beta
PSM: Peptide spectral match
PTEN: Phosphatidylinositol 3,4,5-trisphosphate 3-phosphatase and dual-specificity protein phosphatase PTEN
PTK2: Focal adhesion kinase I
O.C.T.: Optimal cutting temperature
OS: Overall survival
PRKCB: Protein kinase C beta type
RIN: RNA integrity number
RNA-seq: RNA-sequencing
RPM: Reads per million
RPPA: Reverse phase protein microarray
SCNA: Somatic copy number alteration
SDC1: Syndecan I
SRC: Proto-oncogene tyrosine-protein kinase Src
ssGSEA: Single-sample gene set enrichment analysis
TCGA: The Cancer Genome Atlas
TME: Tumor microenvironment
TMT: Tandem mass tags
TP53: Tumor protein 53
UCEC: Uterine corpus endometrial carcinoma
UHR: Universal human reference
USC: Uterine serous carcinoma
VCAN: Versican
