# Supplementary figures and images for: Mapping three-dimensional intratumor proteomic heterogeneity in uterine serous carcinoma by multiregion microsampling
Source: Clin Proteomics. 2024 Jan 22;21:4. doi: 10.1186/s12014-024-09451-2 (PMC10804562; doi:10.1186/s12014-024-09451-2)

## Slide 1
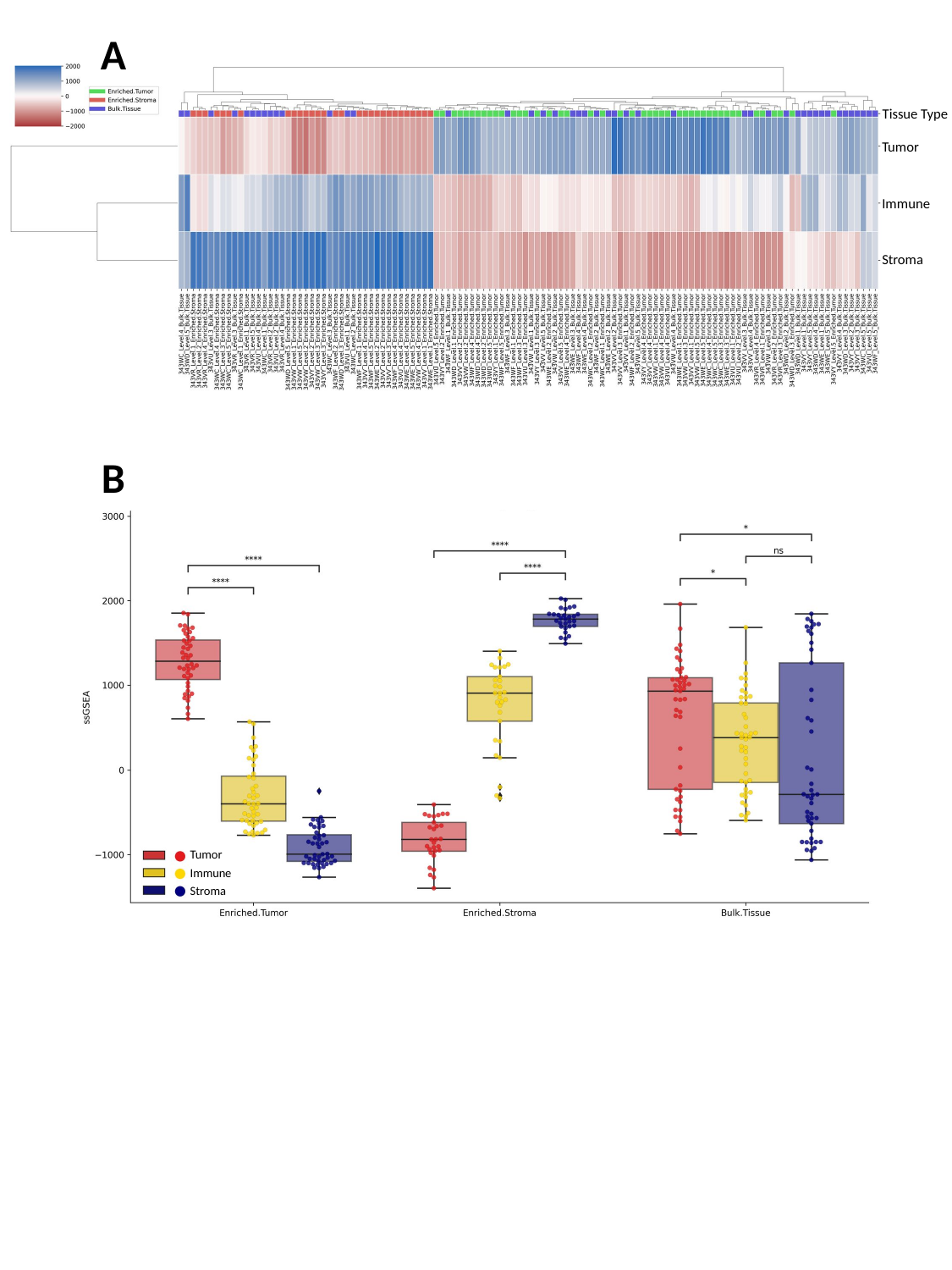

A
Tissue Type
Tumor
Immune
Stroma
B
Tumor
Immune
Stroma

Supplement: Supplementary file 1 — Additional file 1: Figure S1. ProteoMixture [25] ssGSEA scores of ET, ES, and BT samples. A Stacked heatmap depicting ProteoMixture ssGSEA scores for tumor, immune, and stroma in individual sampling levels from ET, ES, and BT harvests. B Boxplots of ssGSEA score enrichment by LMD collection type. Statistically significant differences between collections types are shown with (*) for p<0.01 and (****) for p<0.0001. NS not significant. [file 12014_2024_9451_MOESM1_ESM.pptx]

## Slide 1
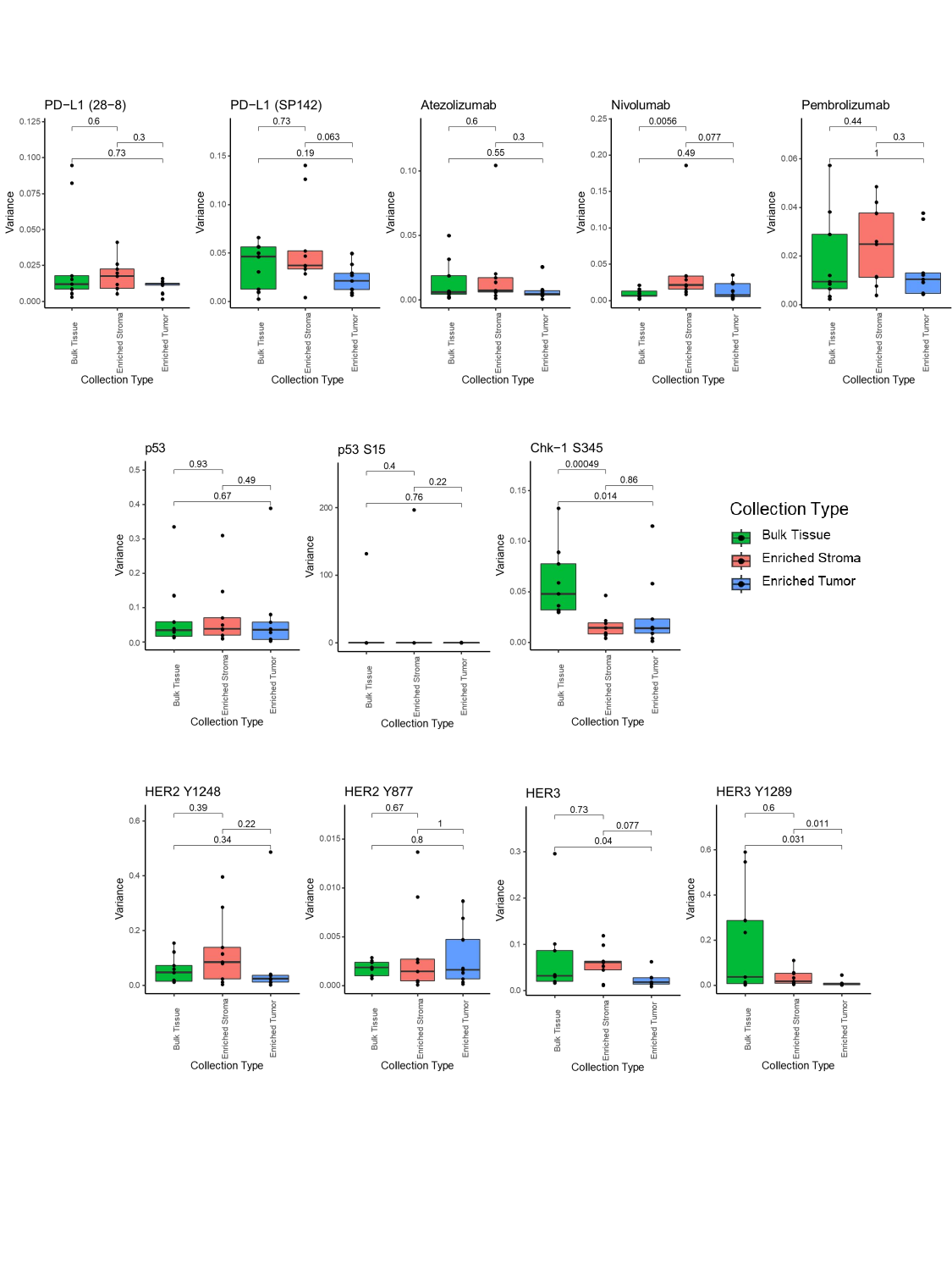

Supplement: Supplementary file 4 — Additional file 4: Figure S4. Variance between sampling levels for each LMD collection type of selected RPPA analytes not quantified by MS. Selected analytes represent biomarkers relevant to ongoing clinical trials enrolling patients with USC and/or other endometrial cancers. [file 12014_2024_9451_MOESM4_ESM.pptx]
