# Supplementary material for: Mapping three-dimensional intratumor proteomic heterogeneity in uterine serous carcinoma by multiregion microsampling
Source: Clin Proteomics. 2024 Jan 22;21:4. doi: 10.1186/s12014-024-09451-2 (PMC10804562; doi:10.1186/s12014-024-09451-2)
Supplement: Supplementary file 2 — Additional file 2: Figure S2. Boxplot depicting relative protein abundance for MUC16. Asterisk (*) indicates significant difference between ES and ET (Wilcox p<0.0001). [file 12014_2024_9451_MOESM2_ESM.pptx]

## Slide 1
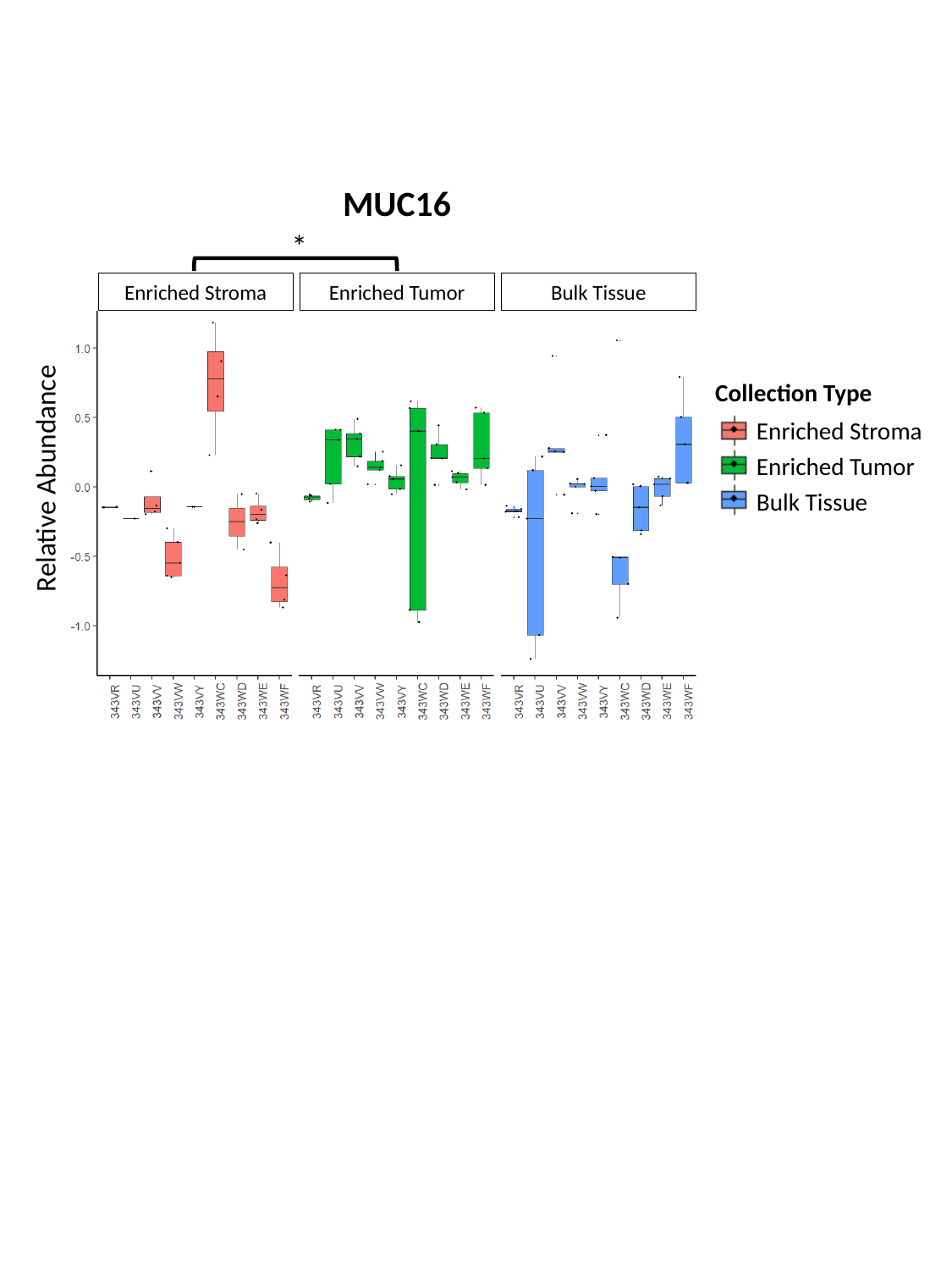

MUC16
*
Enriched Tumor
Bulk Tissue
Enriched Stroma
Collection Type
Enriched Stroma
Enriched Tumor
Bulk Tissue
Relative Abundance
