# Supplementary material for: Mapping three-dimensional intratumor proteomic heterogeneity in uterine serous carcinoma by multiregion microsampling
Source: Clin Proteomics. 2024 Jan 22;21:4. doi: 10.1186/s12014-024-09451-2 (PMC10804562; doi:10.1186/s12014-024-09451-2)
Supplement: Supplementary file 3 — Additional file 3: Figure S3.Protein-RNA Spearman Correlation Matrix for case 343VY. Spearman correlation analysis of 6,019 genes that were co-measured as proteins and corresponding transcripts in 343VY. Size and color of each circle reflects Spearman correlation. [file 12014_2024_9451_MOESM3_ESM.pptx]

## Slide 1
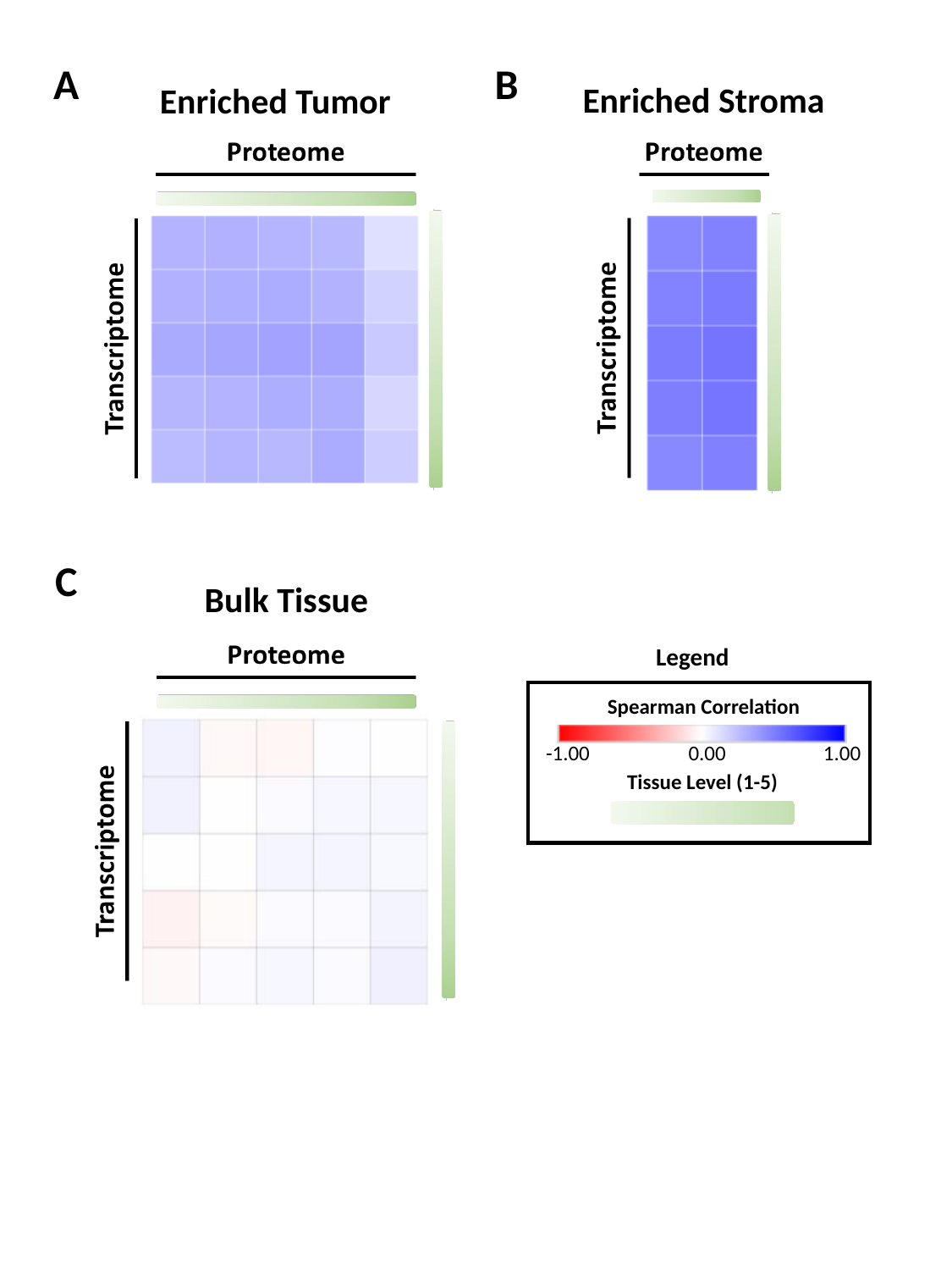

A
B
Enriched Stroma
Enriched Tumor
C
Bulk Tissue
Legend
Spearman Correlation
Tissue Level (1-5)
-1.00
0.00
1.00
