# Supplementary material for: Mapping three-dimensional intratumor proteomic heterogeneity in uterine serous carcinoma by multiregion microsampling
Source: Clin Proteomics. 2024 Jan 22;21:4. doi: 10.1186/s12014-024-09451-2 (PMC10804562; doi:10.1186/s12014-024-09451-2)
Supplement: Supplementary file 5 — Additional file 5: Figure S5. Ridgeline plot of pairwise Spearman correlations between BT harvests per case. The red-white-blue color scale represents Spearman correlations calculated from pairwise comparasions of each BT sampling level for the specified case. The greyscale color of the individual points (n=20 per case) represent the median tumor cellularity. The minimum and maximum values of median tumor cellularity per case are notated on the y-axis. The vertical height of peaks on the y-axis represent the density of the data points (correlations), scaled to 1. [file 12014_2024_9451_MOESM5_ESM.pptx]

## Slide 1
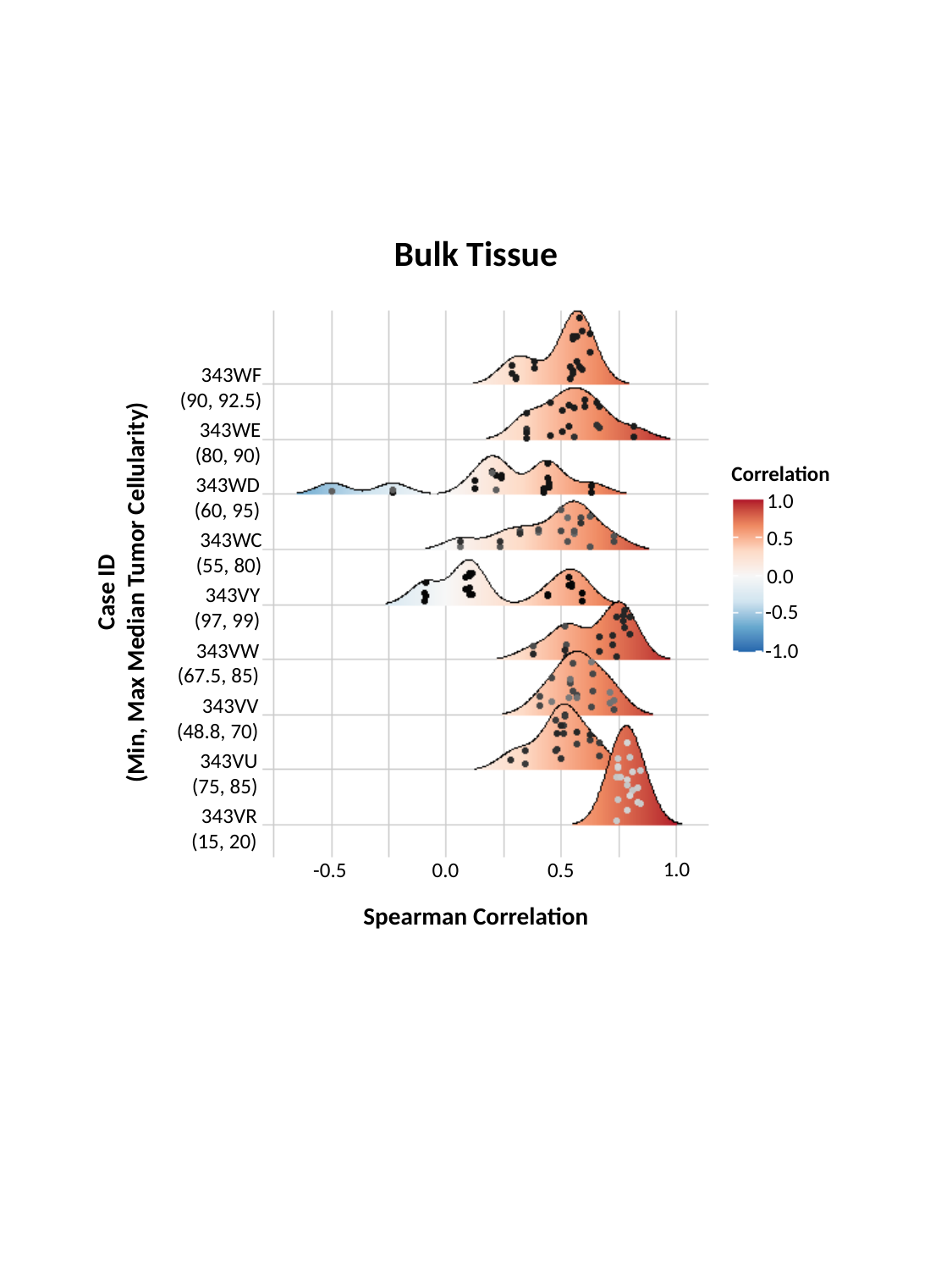

Bulk Tissue
343WF
(90, 92.5)
343WE
(80, 90)
Correlation
1.0
0.5
0.0
-0.5
-1.0
343WD
(60, 95)
343WC
(55, 80)
Case ID
(Min, Max Median Tumor Cellularity)
343VY
(97, 99)
343VW
(67.5, 85)
343VV
(48.8, 70)
343VU
(75, 85)
343VR
(15, 20)
1.0
-0.5
0.0
0.5
Spearman Correlation
