# Supplementary material for: Mapping three-dimensional intratumor proteomic heterogeneity in uterine serous carcinoma by multiregion microsampling
Source: Clin Proteomics. 2024 Jan 22;21:4. doi: 10.1186/s12014-024-09451-2 (PMC10804562; doi:10.1186/s12014-024-09451-2)
Supplement: Supplementary file 6 — Additional file 6: Figure S6. Boxplots depicting relative protein abundances for SOX9 and KRT23. Asterisks indicate significant difference between ES and ET. A single asterisk (*) represents Wilcox p<0.05; double asterisks (**) represent Wilcox p<0.0001. [file 12014_2024_9451_MOESM6_ESM.pptx]

## Slide 1
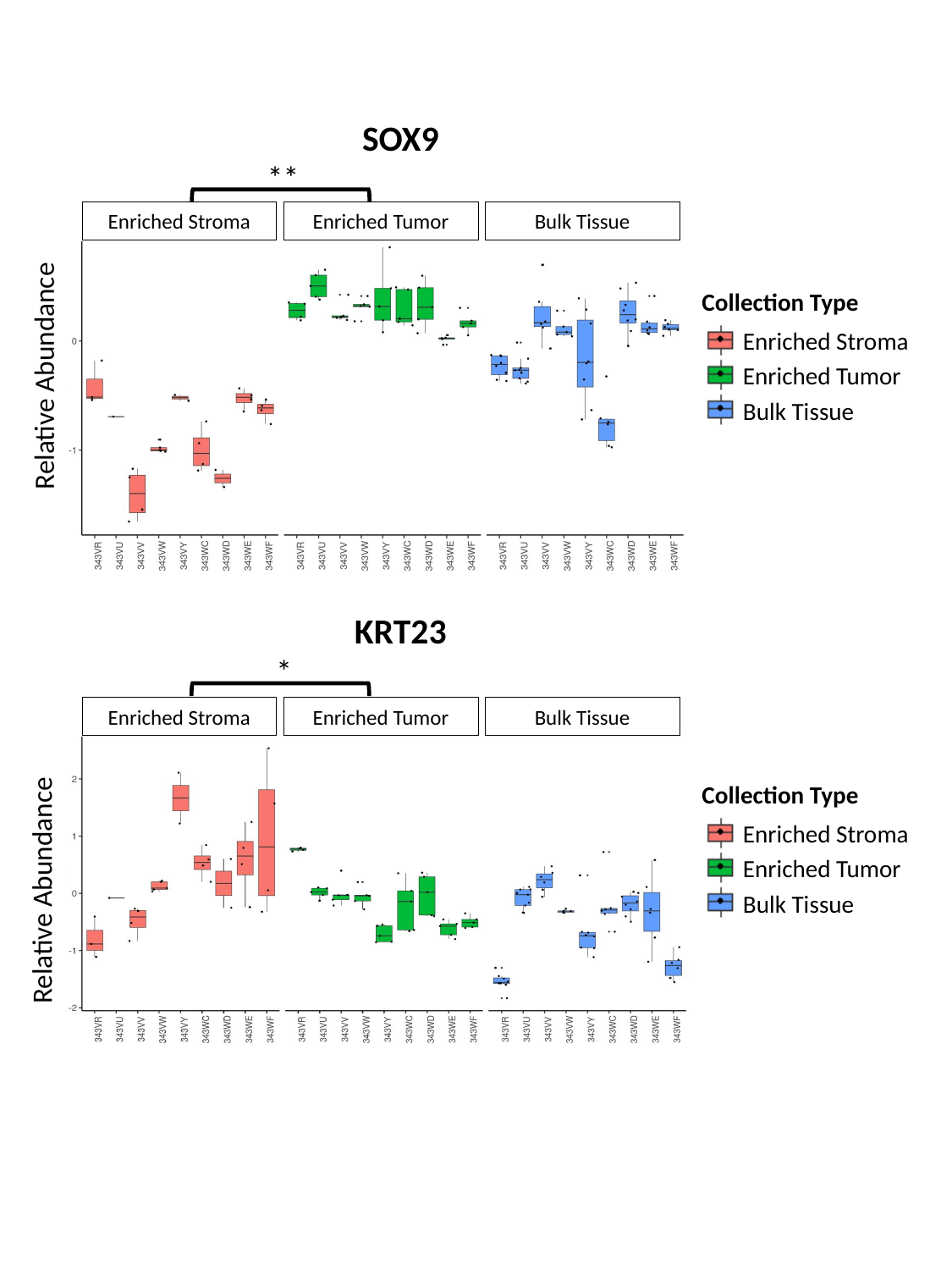

SOX9
**
Enriched Tumor
Bulk Tissue
Enriched Stroma
Collection Type
Enriched Stroma
Enriched Tumor
Bulk Tissue
Relative Abundance
KRT23
*
Enriched Tumor
Bulk Tissue
Enriched Stroma
Collection Type
Enriched Stroma
Enriched Tumor
Bulk Tissue
Relative Abundance
